# 3D Metal–Organic Frameworks Based on Co(II) and Bithiophendicarboxylate: Synthesis, Crystal Structures, Gas Adsorption, and Magnetic Properties

**DOI:** 10.3390/molecules26051269

**Published:** 2021-02-26

**Authors:** Vadim A. Dubskikh, Anna A. Lysova, Denis G. Samsonenko, Alexander N. Lavrov, Konstantin A. Kovalenko, Danil N. Dybtsev, Vladimir P. Fedin

**Affiliations:** Nikolaev Institute of Inorganic Chemistry, Siberian Branch of the Russian Academy of Sciences, 630090 Novosibirsk, Russia; dubskikh@niic.nsc.ru (V.A.D.); lysova@niic.nsc.ru (A.A.L.); denis@niic.nsc.ru (D.G.S.); lavrov@niic.nsc.ru (A.N.L.); k.a.kovalenko@niic.nsc.ru (K.A.K.); cluster@niic.nsc.ru (V.P.F.)

**Keywords:** cobalt(II), metal–organic framework, 2,2′-bithiophen-5,5′-dicarboxylic acid, gas adsorption, adsorption selectivity, magnetic measurements

## Abstract

Three new 3D metal-organic porous frameworks based on Co(II) and 2,2′-bithiophen-5,5′-dicarboxylate (btdc^2−^) [Co_3_(btdc)_3_(bpy)_2_]·4DMF, **1**; [Co_3_(btdc)_3_(pz)(dmf)_2_]·4DMF·1.5H_2_O, **2**; [Co_3_(btdc)_3_(dmf)_4_]∙2DMF∙2H_2_O, **3** (bpy = 2,2′-bipyridyl, pz = pyrazine, dmf = *N*,*N*-dimethylformamide) were synthesized and structurally characterized. All compounds share the same trinuclear carboxylate building units {Co_3_(RCOO)_6_}, connected either by btdc^2–^ ligands (**1**, **3**) or by both btdc^2–^ and pz bridging ligands (**2**). The permanent porosity of **1** was confirmed by N_2_, O_2_, CO, CO_2_, CH_4_ adsorption measurements at various temperatures (77 K, 273 K, 298 K), resulted in BET surface area 667 m^2^⋅g^−1^ and promising gas separation performance with selectivity factors up to 35.7 for CO_2_/N_2_, 45.4 for CO_2_/O_2_, 20.8 for CO_2_/CO, and 4.8 for CO_2_/CH_4_. The molar magnetic susceptibilities χ_p_(*T*) were measured for **1** and **2** in the temperature range 1.77–330 K at magnetic fields up to 10 kOe. The room-temperature values of the effective magnetic moments for compounds **1** and **2** are μ_eff_ (300 K) ≈ 4.93 μ_B_. The obtained results confirm the mainly paramagnetic nature of both compounds with some antiferromagnetic interactions at low-temperatures *T* < 20 K in **2** between the Co(II) cations separated by short pz linkers. Similar conclusions were also derived from the field-depending magnetization data of **1** and **2**.

## 1. Introduction

Metal–organic frameworks (MOFs) are a class of materials consisting of metal ions or polynuclear complexes connected by polytopic organic ligands into regular periodic networks with internal pores. Such materials can be utilized in various important applications, such as gas storage, molecular separation, luminescence, and catalysis. Magnetic properties are also one of the most intriguing characteristics of MOFs assembled from the paramagnetic metal cations or, very rarely, using paramagnetic organic linker [1,2,3,4,5]. The magnetic interactions between the paramagnetic centers of the coordination framework often take place along organic ligands with conjugate double or triple bonds which provide substantial electron delocalization. Carboxylate linkers, such as terephthalate or trimesate, dominate in the current MOF chemistry because of their intrinsic structural rigidity and strong coordination abilities towards common transition metal cations. Such type of ligands can also support the magnetic interaction between the paramagnetic centers; however, the corresponding constants of the magnetic exchange in e.g., terephthalate-based MOFs are, typically, rather low [6,7,8]. Recently several research groups synthesized a number of MOFs using rigid thiophene- and selenophene-based heterocyclic carboxylate ligands [9,10,11,12,13]. The introduction of such heterocyclic aromatic moieties was shown to remarkably improve the adsorption properties of the MOFs [14,15,16,17,18] and also enhances their luminescence [19,20,21,22,23,24]. Moreover, the electron-rich S heteroatom increases the electron density of the thiophene aromatic system which may improve the corresponding magnetic exchange and provide new promising multifunctional materials. Therefore, the synthesis of new MOFs with permanent pores, paramagnetic metal cations and thiophene-based bridging ligands as well as systematic investigation of their functional properties have both academic significance and practical importance. Herein, we report the synthesis and crystal structures of three new 3D metal-organic frameworks based on Co(II) and 2,2′-bithiophen-5,5′-dicarboxylate anionic ligand rarely used in MOF chemistry. The compounds are characterized by a number of analytical methods, including N_2_, O_2_, CO, CO_2_, CH_4_ gas adsorption isotherms at various conditions. The magnetic properties were also measured and rationalized towards their crystal structures.

## 2. Results and Discussion

### 2.1. Synthesis and Structural Characterization

Three metal-organic frameworks [Co_3_(btdc)_3_(bpy)_2_]·4DMF (**1**, bpy = 2,2′-bipyridyl), [Co_3_(btdc)_3_(pz)(dmf)_2_]·4DMF·1.5H_2_O (**2**, pz = pyrazine), and [Co_3_(btdc)_3_(dmf)_4_]∙2DMF∙2H_2_O (**3**) were crystallized in very similar reaction conditions using Co(II) salt and 2,2′-bithiophen-5,5′-dicarboxylatic acid (H_2_btdc) at the same concentrations in N,N-dimethylformamide (DMF). Some minor variations of the temperature or anion nature were necessary to improve the crystallinity of the product. Unfortunately, compound **3** was always contaminated by some unknown amorphous precipitate; therefore, its chemical composition and crystal structure were established by the single-crystal X-ray diffraction method. The color of the crystals **1**–**3** (purple or red) suggests the retention of the +2 oxidation state of the Co(II) cations in each compound, which was further confirmed by the analysis of the interatomic distances and magnetic measurements (vide infra).

**Figure 1:**
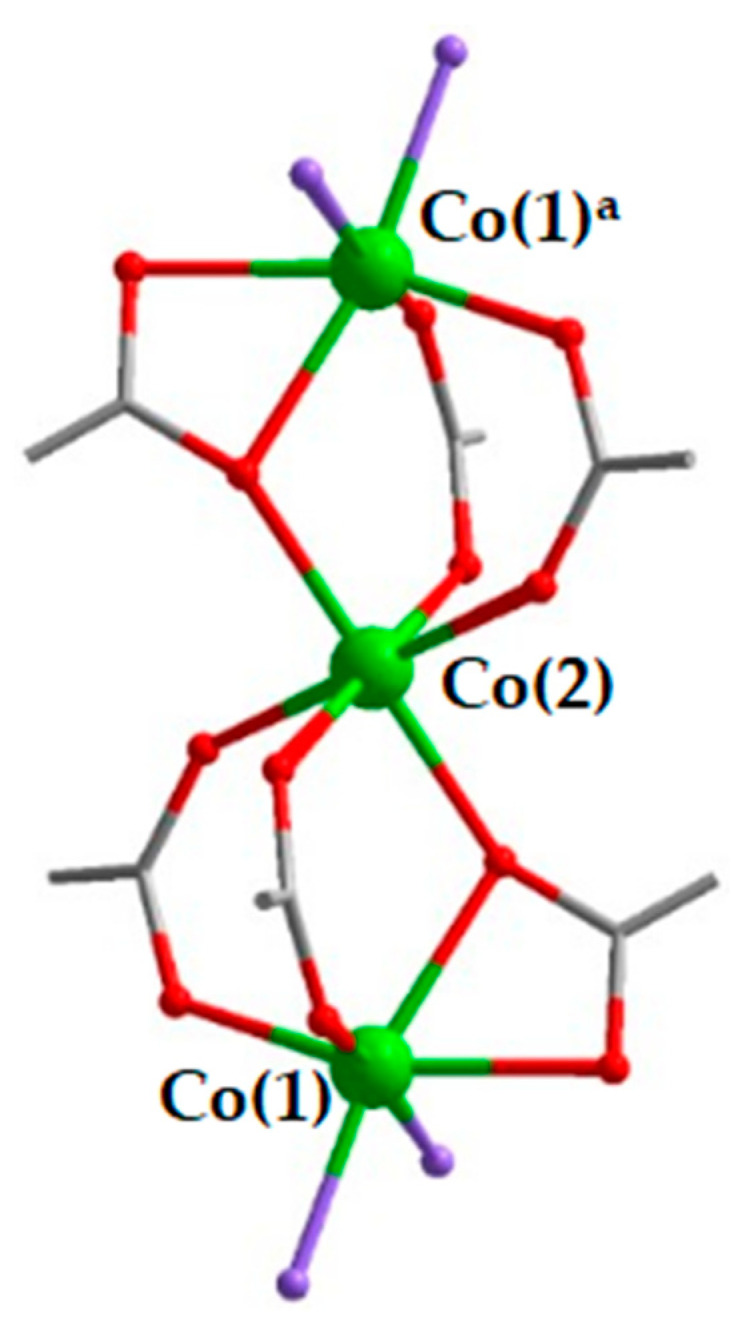
Structure of the {Co_3_(RCOO)_6_} building unit in **1**–**3**. Co–green; O–red; C–grey. Purple atoms indicate either O atoms of the dmf solvent molecules and/or N atoms of the auxiliary ligands (2,2′-bpy, pz). ^a^ generated by inversion center.

The structural analyses reveal that all three MOF compounds are made of the same principle unit in their coordination structure. It contains three linearly aligned Co(II) cations, connected by six carboxylate groups, four in bridging mode, two in bridging/chelating mode: three carboxylate anions for each pair of cations (Figure 1). The central Co(2) is in centro-symmetrical octahedral coordination of O atoms of six carboxylate groups. The terminal Co(1) cations are in distorted octahedral coordination, composed of four O atoms of three carboxylate groups and two other atoms (either O or N) of solvent molecules or auxiliary ligands, depending on each particular case. The resulting trinuclear {Co_3_(μ-RCOO-κ^1^,κ^1^)_4_(μ-RCOO-κ^1^,κ^2^)_2_} units are rather common motives in the crystal structures of MOFs based on certain s- and d- M(II) metals, such as Mg, Zn, Mn, Co, Cd [25,26,27,28,29,30,31]. Very likely, the persistency of such {Co_3_(RCOO)_6_} building unit in all the title compounds **1**–**3** results from the similarity of their reaction conditions, which directs the self-assembly along the same general route: the equimolar amounts of Co(II) salt and H_2_btdc form charge-neutral intermediates {Co_3_(RCOO)_6_}, where two vacant coordination positions on each outside Co(II) cation are occupied by auxiliary ligands (2,2′-bpy in **1**; pz and dmf in **2**) when such chemicals are available in the reaction mixture and/or by solvent molecules in the absence of the N-donor ligands (dmf in **3**).

**Figure 2:**
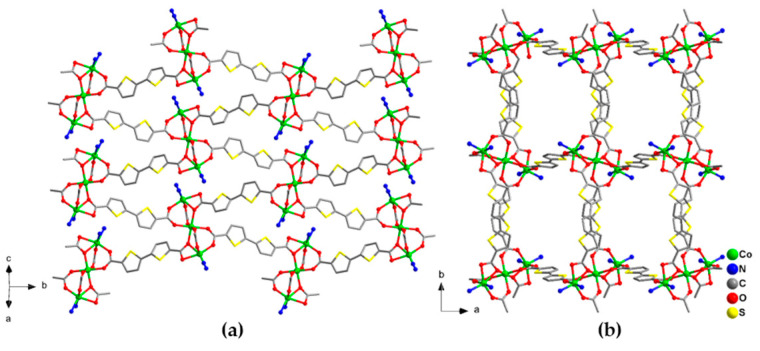
Projection of the crystal structure of **1** along different directions. Cobalt atoms are shown by green balls. Only N atoms of coordinated bpy ligands are shown. Hydrogen atoms and guest solvent molecules are omitted.

Purple stick crystals of [Co_3_(btdc)_3_(bpy)_2_]·4DMF (**1**) are obtained by heating of mixture of Co(NO_3_)_2_·6H_2_O, H_2_btdc and 2,2′-bpy in 2:2:1 molar ratio in DMF at 110 °C during 2 days. According to the single-crystal X-ray diffraction data, compound **1** crystallizes in the monoclinic space group *P*2_1_/*c*. Two additional positions on each Co(1) cations of the {Co_3_(RCOO)_6_} building units are occupied by one chelating 2,2′-bpy ligands forming [Co_3_(RCOO)_6_(bpy)_2_] complexes (Figure S1a). Within such complexes the Co(1)–O bond lengths are in the range 2.0110(2)–2.209(2) Å, the Co(1)–N distances are 2.079(2) Å and 2.131(3) Å, the Co(2)–O distances lay in the range 2.042(2)–2.1209(18) Å, and the Co(1)–Co(2) interatomic distances are 3.5282(7) Å. Each {Co_3_(RCOO)_6_} unit in **1** is connected by bridging btdc^2–^ ligands to six others thus serving as a 6-connected node of the regular coordination framework with underlying primitive-cubic topology **pcu** (Figure 2). The average distances between the {Co_3_(RCOO)_6_} units in the structure are, ca. 14 Å, according to the size of the rather long btdc^2–^ anions. Despite rather strong distortions, the resulting framework **1** possesses rectangular channels of 6 × 5 Å along the *c* crystallographic axis, filled with the solvent DMF molecules (Figure 2b). The guest accessible volume of **1**, calculated using PLATON software [32], is 35%. The guest composition was established from multiple methods, including SQUEEZE analysis of the unassigned electron density, IR spectroscopy, thermogravimetric and chemical analyses.

**Figure 3:**
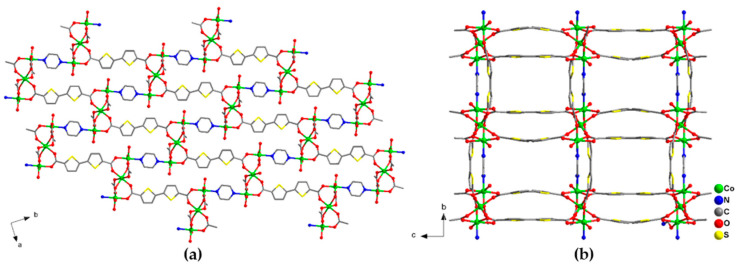
Projection of the crystal structure of **2** along different directions. Cobalt atoms are shown by green balls. Only O atoms of coordinated dmf ligands are shown. Hydrogen atoms and guest solvent molecules are not shown.

Red stick crystals [Co_3_(btdc)_3_(pz)(dmf)_2_]·4DMF·1.5 H_2_O (**2**) are formed by heating the Co(ClO_4_)_2_·6H_2_O, H_2_btdc and pyrazine mixture in DMF at 105 °C for two days. Some amount (10 μL) of concentrated hydrochloric acid was added to the reaction solution in order to dissolve the pyrazine. According to the single-crystal X-ray diffraction data, compound **2** crystallizes in the orthorhombic space group *Fddd*. The two additional positions on Co(1) cations in **2** are occupied by one O atom of the coordinated dmf solvent molecule and one N atom of the bridging pyrazine molecule, which connects two Co(1) cations of the neighboring trinuclear complexes [Co_3_(btdc)_3_(pz)(dmf)_2_] (Figure S2). The interatomic Co(1)–O bond distances are in the range 2.0129(14)–2.1732(15) Å, the Co(1)–N distance is 2.1497(17) Å, the Co(2)–O bond lengths lay in the range 2.0737(14)–2.1520(13) Å, and the interatomic Co(1)–Co(2) distances in the trinuclear complexes are 3.4829(4) Å. Contrary to **1**, the carboxylate {Co_3_(RCOO)_6_} units in framework **2** are interconnected by both btdc^2–^ and pz bridging ligands in three directions (Figure 3). Despite a higher number of potential linkers, the connectivity of the {Co_3_(btdc)_3_(pz)} complexes in **2** is 6 because four btdc^2–^ ligands form two double bridges, which results in a complex uninodal 6-connected topology of the framework with point symbol 4^10^.6^5^. To the best of our knowledge, such topology has never been mentioned or characterized in the literature. There are three types of distances between the thinuclear carboxylate units {Co_3_(RCOO)_6_} in the crystal structure **2**: 13.7 Å for the single btdc^2–^ bridge, 13.3 Å for the double btdc^2–^ bridge, and 10.9 Å for the pz bridge (measured between the central Co(2) cations) with minimal Co–Co spacing being as short as 7.1 Å (measured between Co(1) cations). The framework structure features rectangular channels along the a-axis of 7 × 4 Å, filled by guest DMF and H_2_O molecules (Figure 3b). The free accessible volume is ca. 39% [32]. The guest composition was established from the multiple instrumental methods, such as SQUEEZE analysis, IR spectroscopy, TGA, chemical analyses.

**Figure 4:**
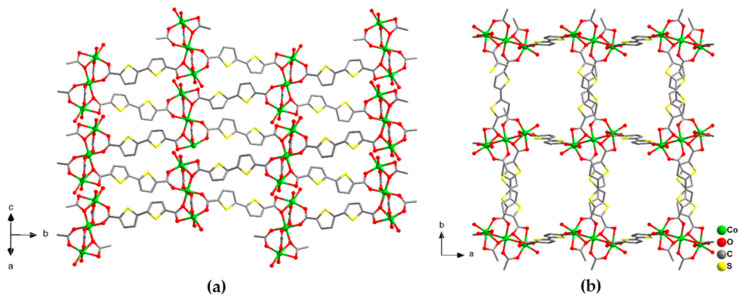
Projection of the crystal structure of **3** along with different directions (**a**,**b**). Cobalt atoms are shown by green balls. Only O atoms of coordinated dmf ligands are shown. Hydrogen atoms and guest solvent molecules are omitted.

Purple block crystals of [Co_3_(btdc)_3_(dmf)_4_]·2DMF·2H_2_O (**3**) are obtained by heating of an equimolar mixture of Co(NO_3_)_2_·6H_2_O and H_2_btdc b DMF at 110 °C for two days. According to the single-crystal X-ray diffraction data, compound **3** crystallizes in the monoclinic *P*2_1_/*c* space group and have a very similar structure to **1**. Two additional positions on each Co(1) cations of the {Co_3_(RCOO)_6_} units in **3** are occupied by two positionally disordered dmf solvent molecules (Figure S1b). The Co(1)–O bond length are in the range 1.958(4)–2.197(4) Å, Co(2)–O bond length are in the range 2.064(3)–2.136(5) Å, the Co(1)–Co(2) distances in {Co_3_(RCOO)_6_} building block are 3.5576(42) Å. Similarly to **1**, framework **3** adopts **pcu** topology with rectangular channels of ca. 3 *×* 5 Å in the crystallographic ab plane, filled by guest solvent DMF molecules (Figure 4). The free accessible volume in **3** was estimated to be 33%, assuming the removal of only guest dmf molecules [32].

### 2.2. IR-Spectroscopy, Thermal Activation, and Porosity

The compounds **1** and **2** were obtained as pure phases, as confirmed by powder X-ray diffraction method (Figure S3) and chemical analyses; hence, their functional properties were further characterized.

The IR spectrum of the compound **1** (Figure S4) contains a band at 768 cm^−1^ related to non-planar deformation vibrations of C–H bonds in the thiophene fragment, characteristic bands of carboxylate groups at 1376 cm^−1^ and 1440 cm^−1^ associated with symmetric stretching vibrations, a group of bands in the range from 1477 cm^−1^ to 1597 cm^−1^ originating from skeletal vibrations of aromatic fragments of the ligands. The intense band at 1665 cm^−1^ is related to valence vibrations of the C=O bond in DMF molecules. The band at 3090 cm^−1^ corresponds to stretching vibrations of the C–H bond in the thiophene fragment. The broad band at 3428 cm^−1^ can be attributed to water on the external surfaces of the crystals. The IR spectrum of the compound **2** (Figure S5) contains a band at 771 cm^−1^ correspondings to non-planar deformation vibrations of C–H bonds in the thiophene fragment, strong intensity bands at 1376 cm^−1^ and 1440 cm^−1^ related to the symmetric stretching vibration of carboxylate groups, a group of medium intensity bands in the range from 1517 cm^−1^ to 1589 cm^−1^ associated with skeletal vibrations of the pyrazine molecule and thiophene fragments. The band at 1652 cm^−1^ can be referred to C=O bonds in DMF molecules, whereas the low-intensity bands at 2854 cm^−1^ and 2926 cm^−1^ correspond to the valence vibrations of their C–H bonds. The broad band at 3449 cm^−1^ is related to the valence vibrations of the O–H bond of guest water molecules.

**Figure 5:**
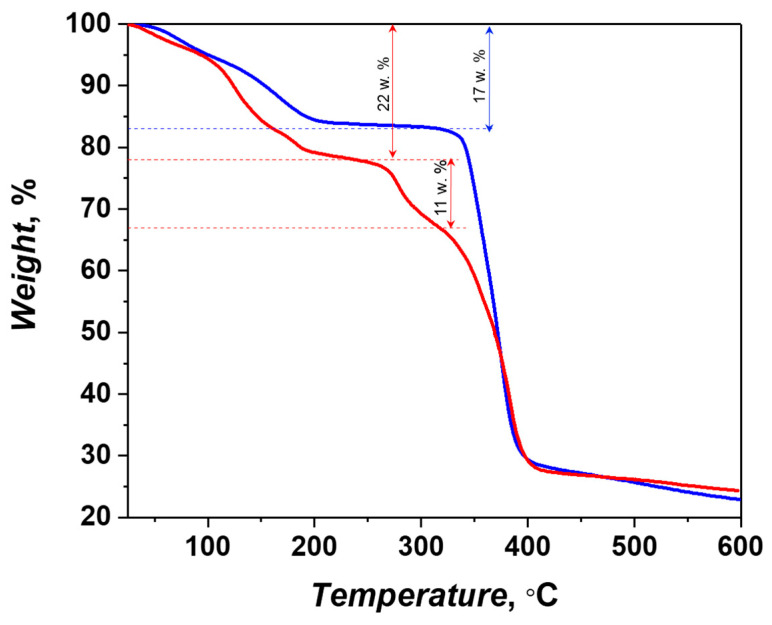
Thermogravimetric curves for compounds **1** (blue) and **2** (red).

The thermogravimetric analysis (Figure 5) of compound **1** reveals a continuous mass loss in a wide temperature range (up to 200 °C) of ca. 17%, which can be ascribed to the removal of the guest solvent molecules (calculated: 19% for 4 DMF), followed by a broad flat region up to ca. 330 °C, where rapid degradation of the metal-organic framework takes place. The heating of compound **2** results in a step-like decrease of the sample weight of ca. 22% up to 230 °C, corresponding the evaporation of the solvent molecules (calculated: 23% for 4DMF + 1.5H_2_O). The second stage of the weight loss of ca. 11% occurs near 280 °C and corresponds to the release of the coordinated DMF molecules (calculated: 10% for 2DMF). The irreversible thermolysis of the metal-organic framework **2** occurs above 340 °C, similarly to that for compound **1**.

**Figure 6:**
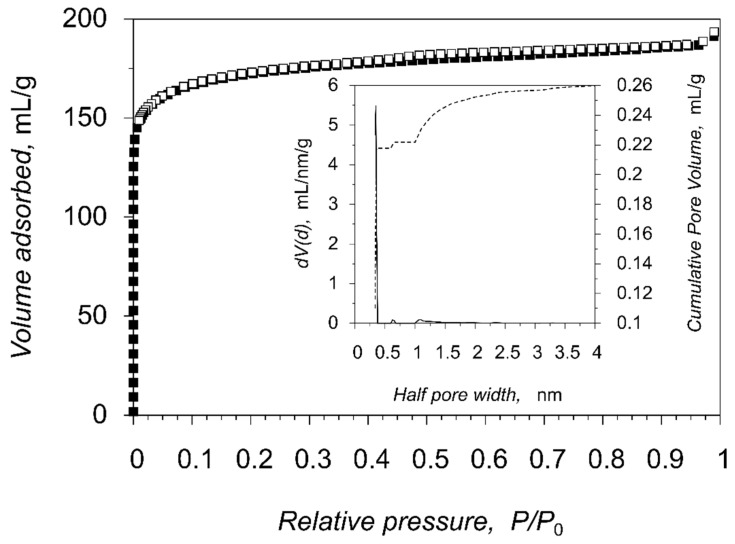
Nitrogen adsorption (filled squares) and desorption (open squares) isotherms at 77 K for compound **1**. The inset shows the pore size distribution.

The permanent porosity was confirmed by the measurements of the gas adsorption for compound **1**, which has a broad temperature stability range and plain activation strategy. The as-synthesized crystals of the porous compound **1** were activated by solvent exchange (CH_2_Cl_2_), followed by a dynamic vacuum treatment at 30 °C for 1 h directly in a gas adsorption analyzer. The nitrogen adsorption-desorption isotherm plots at 77 K are represented in Figure 6, Figures S6 and S7 and belong to the Ia isotherm type according to the official IUPAC classification [33], which is typical for microporous compounds with narrow slit pores. The measured pore volume 0.289 mL g^−1^ (at p/p_0_ = 0.95) matches the expected value (0.280 mL g^−1^) from the PLATON pore volume calculations, which confirms the structural integrity of **1** as well as completeness of the framework activation. The calculated surface areas are 758 m^2^ g^−1^ (Langmuir model), 667 m^2^ g^−1^ (BET model) and 671 m^2^ g^−1^ (DFT model). The distribution of the pore size was calculated using the nonlinear DFT equilibrium model with slit pores which gives good agreement between measured and calculated isotherms. Pore size distribution plot of **1**, calculated by DFT model, (Figure 6, inset) shows the presence of narrow pores of ca. 7 Å. Similarly, the corresponding pore-size distribution, calculated by Zeo++ program package [34,35] on the basis of the structural data of **1** results in the same value of 7 Å (Figure S8), validating both methods and supporting the structural stability of the guest-free framework.

### 2.3. Gas Adsorption Selectivity

**Figure 7:**
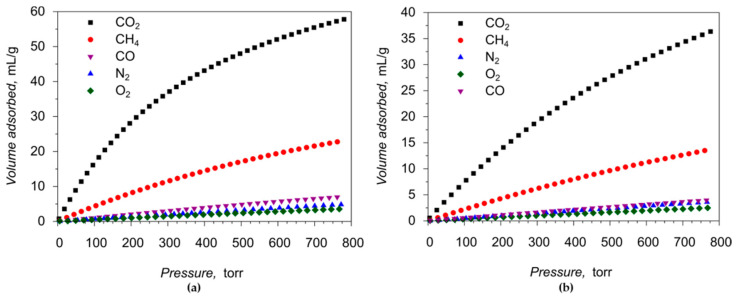
CO_2_, N_2_, O_2_, CO and CH_4_ gas adsorption isotherms for the compound **1**: (**a**) at 273 K; (**b**) at 298 K.

More detailed gas adsorption properties of **1** were further investigated by measuring the adsorption-desorption isotherms for CO_2_, N_2_, CO, O_2_ and CH_4_ at T = 273 and 298 K. The corresponding isotherms obtained are shown in Figure 7, Figures S9 and S11, gas uptakes in different units are summarized in Table 1.

The absolute amounts of the gas uptakes of the porous compound **1** at ambient conditions are moderate, compared with the best results for other MOFs, which is expected of the frameworks with relatively narrow pores and moderate surface area. The calculated isosteric heats of adsorption at zero coverage Q_st_(0) are 26.1 kJ⋅mol^−1^ for CO_2_, 19.7 kJ⋅mol^−1^ for CH_4_, 17.3 kJ⋅mol^−1^ for CO, 10.3 kJ⋅mol^−1^ for O_2_, and 8.8 kJ⋅mol^−1^ for N_2_ (Figure S10). Such low values are typical for the physical adsorption through weak van-der-Waals interactions and consistent with the structural data of **1**, with all coordination positions near the Co(II) cations being saturated by either carboxylate groups or chelated bpy ligand. We note here that, from the practical application point of view, a low value of the adsorption heat is highly beneficial since it reduces the energy penalties in the cyclic temperature-swing adsorption separation processes.

The porous materials with a narrow diameter of pores usually exhibit great gas adsorption selectivity. The selectivity factors for separation of CO_2_/N_2_ and CO_2_/CH_4_ binary gas mixtures on **1** were calculated by three different methods: (i) as the ratio of the amount adsorbed; (ii) as the ratio of corresponding Henry constants; and (iii) by Ideal Adsorbed Solution Theory (IAST) [36] calculations, which allows estimation of the selectivity factors at different compositions of the gas mixture and various total pressures. The results are summarized in Table 2 and in Figure S12. Depending on the method used, the adsorption selectivity factors of CO_2_/N_2_ gas mixture vary from 10 to 35.7 at 273 K and from 7.9 to 18.9 at 298 K, which is well above average, considering other porous MOFs with no coordinatively unsaturated metal sites (CUS). The IAST CO_2_/N_2_ selectivity values become even greater for the gas mixture composition similar to the real industrial flue gases (CO_2_:N_2_ = 15:85 (v/v), Table 2). The CO_2_/O_2_ adsorption selectivity factors range from 9.4 to 45.4, depending on the temperature and calculation approach, similarly to results reported for other porous MOFs [37,38,39]. The obtained adsorption selectivity factors for the CO_2_/CO mixture (7.9 ÷ 20.8 at 273 K, 7.2 ÷ 15.1 at 298 K) rival the most prominent results reported for porous MOFs with only a few superior exceptions [40,41,42]. More importantly, all the obtained selectivity factors are in par or exceed 8, which is considered to be sufficient for a practical separation of gases [43]. The adsorption selectivity factors for CO_2_/CH_4_ mixture range from 2.5 to 4.8, depending on the temperature and calculation method used. Such values are comparable to many reported porous MOF compounds [38,44,45]. Appreciable CO_2_/N_2_, CO_2_/CO and CO_2_/O_2_ separation factors, as well as low CO_2_ adsorption heat, put the porous material **1** among other promising platforms for the development of efficient processes for the sequestration of CO_2_ from N_2_, CO or O_2_, which addresses important environmental, industrial and safety challenges, respectively.

### 2.4. Magnetic Properties of Compounds ***1*** and ***2***

**Figure 8:**
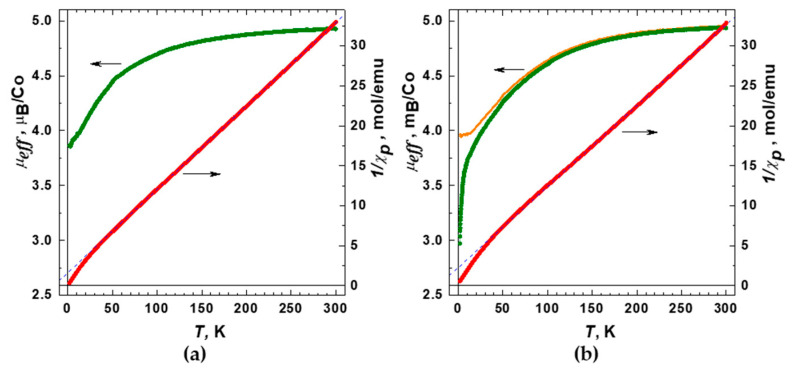
Temperature dependences of the reciprocal magnetic susceptibility 1/χ_p_ (paramagnetic component) and effective magnetic moment µ_eff_ per one cobalt ion for the compound: (**a**) **1**; (**b**) **2**. Dashed blue lines indicate formal Curie–Weiss fits for the high-temperature region. The orange curve in plot (**b**) shows the µ_eff_ data obtained by taking into account the antiferromagnetic intermolecular interactions. Magnetic field 10^3^ Oe.

Temperature dependences of the molar magnetic susceptibility were measured for **1** and **2** in the range 1.77–330 K at magnetic fields up to 10 kOe under zero-field cooled and field-cooled conditions (Figure S13). The data obtained revealed no dependence on the magneto-thermal history implying the absence of any ferromagnetic or spin-freezing phenomena. For both compounds, **1** and **2**, the magnetic susceptibility increased gradually with the decreasing temperature down to the lowest accessed temperature of 1.77 K, as expected for paramagnetic substances, such as MOF containing magnetic Co(II) ions. After subtracting the diamagnetic contribution, χ_d_, calculated using Pascal’s additive scheme, from the total magnetic susceptibility, the remaining paramagnetic component of the molar magnetic susceptibility, χ_p_(T), was plotted as 1/χ_p_ vs. T (Figure 8). As can be seen, the 1/χ_p_(T) curves are quite close to linear at temperatures above ~50 K, nominally following a paramagnetic Curie–Weiss dependence χ_p_(T) = C/(T−θ) with θ ≈ −16 K and −22 K for **1** and **2**, respectively, in close similarity to the behavior observed for other MOFs containing Co(II) [46,47,48]. Given the absence of long-range antiferromagnetic (AFM) ordering and the deviation of the 1/χ_p_ data from the fits towards smaller values (larger values of χ_p_) observed at low temperatures, the linear 1/χ_p_(T) dependences at T > 50 K are rather accidental; hence, the apparent θ values should not be taken as an indicator of magnetic interactions between cobalt ions belonging to neighboring molecules (J′). In fact, much smaller θ values would be obtained if fitting were performed in the lowest temperature region (Figure 8), pointing therefore to a small actual J′.

In order to clarify the observed magnetic behavior, we first focused at the intramolecular magnetic state of Co(II) ions in trinuclear carboxylate {Co_3_(RCOO)_6_} units and calculated the effective magnetic moment μ_eff_ using the Curie dependence (Figure 8). The corresponding equation for one independent cobalt ion is χ_p_(T) = N_a_ μ_eff_^2^/3⋅k_B_⋅T, while for the formula unit, containing a trinuclear complex, it is χ_p_(T) = 3⋅N_a_ μ_eff_^2^/3⋅k_B_⋅T. Both compounds **1** and **2** turn out to have the same μ_eff_(300 K) ≈ 4.93 μ_B_, which exceeds the spin-only value (3.87 μ_B_) for the isolated high-spin Co(II) ions with S = 3/2, implying a significant orbital contribution. Except for the low-temperature range T < 20 K, the temperature dependence of μ_eff_ for **1** and **2** is virtually identical: with decreasing temperature the μ_eff_ is gradually reduced down to ~4 μ_B_ owing to a combination of single-ion zero-field splitting effects (partial quenching of orbital moments) and antiferromagnetic interactions of Co(II) ions within the trimers mediated by the carboxylate bridges. This behavior is rather close to those reported for other similar Co(II) MOFs [49,50,51,52]. Given the complicated magnetic ground state of the octahedrally coordinated Co(II) ions [53], a quantitative analysis of the χ_p_(T) data and exact determination of the AFM exchange interaction of cobalt ions within trimers (J) is hardly achievable. However, the data obtained for two compounds, **1** and **2**, that have similar magnetic state of the trinuclear units and differ in the way they are arranged in the MOFs, give us a possibility to shed light on the weak intermolecular interaction. In case of **1** (Figure 8a), the zero-T extrapolation gives 1/χ_p_(T) = 0 (μ_eff_ ~ 3.7 μ_B_) within the experimental accuracy, implying an almost ideal paramagnetic behavior of Co(II)-trimers non-interacting with each other. Indeed, in structure **1**, all trinuclear complexes [Co_3_(RCOO)_6_(bpy)_2_] are linked by btdc^2–^ ligands only, providing a rather long separation between the {Co_3_(RCOO)_6_} units at ~ 14 Å (vide supra).

**Figure 9:**
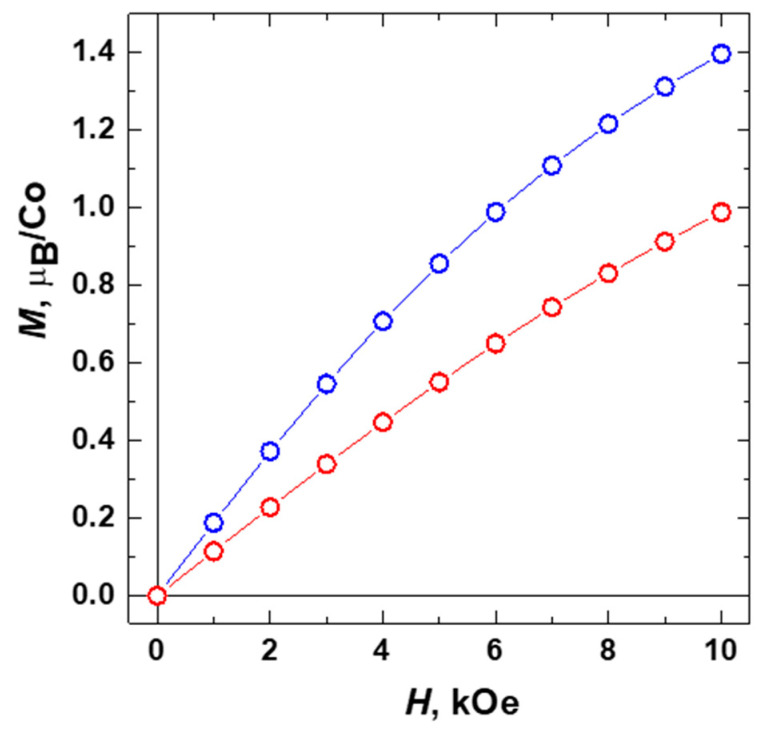
Field-depending magnetization M(H) data for compounds **1** (blue) and **2** (red) taken at T = 1.77 K.

Upon lowering the temperature below 20 K the effective magnetic moment of **2** μ_eff_ drops increasingly fast, which markedly differs from the behavior of **1** (Figure 8). Such notable discrepancies in the magnetic properties likely suggest an additional AFM interaction that comes into play in **2** at low T. We should note here that an introduction of a certain Weiss constant θ = −1.5 K into the expression χ_p_(T) = 3⋅N_a_⋅μ_eff_^2^/3⋅k_B_⋅(T − θ) allows fitting the low-T behavior of **2** and bringing the μ_eff_(T) curve to exactly the same shape as in **1** (Figure 8b, orange curve). It supports the above assumption that the differences in magnetic properties of **1** and **2** originate from the presence of the AFM interactions between the {Co_3_(RCOO)_6_} units in **2**. Even though such interactions are insufficient for a long-range AFM ordering in the observed temperature range, the corresponding trend is clearly manifested in the field-dependence of the magnetization M(H) data (Figure 9), which show noticeably lower values of the magnetization and slower approaching to the spin-polarized state for the compound **2** than that for **1**. Apparently, the emergence of intermolecular AFM interactions in **2** results from a small pyrazine linker, which provides a relatively short connection between the {Co_3_(RCOO)_6_} units at 7.1 Å thus giving rise to some long-range magnetic ordering.

## 3. Materials and Methods

### 3.1. Instruments and Methods

Infrared spectra of solid samples as KBr pellets were recorded using an IR-Fourier spectrometer Scimitar FTS 2000 (4000–400 cm^−1^). The elemental analyses were obtained on an analyzer «Vario Micro-Cube». The thermogravimetric analyses were carried out in He atmosphere on NETZSCH TG 209 F1 thermoanalyzer with a heating rate of 10 deg/min. The powder X-ray diffraction data were obtained on a «Shimadzu XRD 7000S» powder diffractometer (Cu-Kα irradiation, λ = 1.54178 Å). Surface area and porous structure were analyzed using the nitrogen adsorption technique on a Quantochrome’s Autosorb iQ gas sorption analyzer at 77 K. Prior to the isotherm measurements the as-synthesized crystals of compound **1** were placed in CH_2_Cl_2_ for five days. Each day the crystals were separated from the solvent by decantation, and a new portion of CH_2_Cl_2_ was added. After 5 days the crystals were separated from the solvent, placed into the gas-measurement cell and, finally, activated under a dynamic vacuum at 30 °C for 1 h. The nitrogen adsorption–desorption isotherms were measured within the range of relative pressures from 10^−6^ to 0.995. The specific surface area was calculated from the data obtained using the conventional BET and DFT models. Gases adsorption experiments at 273 and 298 K were carried out volumetrically on Quantochrome’s Autosorb iQ equipped with thermostat TERMEX Cryo-VT-12 to adjust the temperature with 0.1 K accuracy. Adsorption–desorption isotherms were measured within the range of pressures of 1 to 800 torr. The database of the National Institute of Standards and Technology available at http://webbook.nist.gov/chemistry/fluid/ was used as a source of p–V–T relations at experimental pressures and temperatures. Magnetization measurements were carried out using a Quantum Design MPMS-XL SQUID magnetometer in the temperature range 1.77–330 K at magnetic fields up to 10 kOe. Temperature dependences of the magnetization, M(T), were measured on heating the sample after it was cooled either in zero magnetic field or in a given magnetic field as well as upon cooling the sample. In order to determine the paramagnetic component of the molar magnetic susceptibility, χ_p_(T), the temperature-independent diamagnetic contribution, χ_d_, and a possible magnetization of ferromagnetic micro-impurities, χ_FM_(T), were evaluated and subtracted from the measured values of the total molar susceptibility χ = M/H. While χ_d_ was calculated using Pascal’s additive scheme, χ_FM_(T), if any, was determined from the measured isothermal M(H) dependencies and the M(T) data taken at different magnetic fields. To determine the effective magnetic moment of cobalt Co(II) ions, µ_eff_, the paramagnetic susceptibility χ_p_(T) was analyzed using the Curie–Weiss dependence χ_p_(T) = N_a_ μ_eff_^2^/3⋅k_B_⋅(T − θ), where N_A_ and k_B_ are the Avogadro and Boltzmann numbers, respectively.

### 3.2. Single-Crystal X-ray Diffraction

Diffraction data for single-crystals **1**–**3** were collected on the ‘Belok’ beamline (λ = 0.793127 Å, φ-scans with a step of 1.0) of the National Research Center ‘Kurchatov Institute’ (Moscow, Russian Federation) using a Rayonix SX165 CCD detector. The data were indexed, integrated and scaled, and an absorption correction was applied using the XDS program package [54]. The structures were solved by the dual space algorithm (SHELXT [55]) and refined by the full-matrix least-squares technique (SHELXL [56]) in the anisotropic approximation (except hydrogen atoms). Positions of hydrogen atoms of organic ligands were calculated geometrically and refined in the riding model. The final compositions of compounds **2** and **3** were defined according to the PLATON/SQUEEZE procedure [32] (2787 e^−^ in 9986 Å^3^ for **2**, 195 e^−^ in 967 Å^3^ for **3**) and the data of elemental (C, H, N, S) analyses. The crystallographic data and details of the structure refinements are summarized in Table S1. CCDC 2023586-2023588 contains the supplementary crystallographic data for this paper. These data can be obtained free of charge from The Cambridge Crystallographic Data Center at https://www.ccdc.cam.ac.uk/structures/.

### 3.3. Synthesis of Coordination Polymers

The ligand synthesis was carried out according to the known methodology [10]. The starting substances were used as commercially available reagents without further purification.

Synthesis of [Co_3_(btdc)_3_(bpy)_2_]∙4DMF (**1**). Cobalt(II) nitrate hexahydrate (29.7 mg, 0.1 mmol), 2,2′-bithiophen-5,5′-dicarboxylic acid (H_2_btdc, 25.4 mg, 0.1 mmol), 2,2′-bipyridine (bpy, 9.0 mg, 0.05 mmol) and 5 mL of *N*,*N*-dimethylformamide (DMF) were placed in a glass vial with a screw cap. The reaction mixture was sonicated for 30 min, and then heated at 110 °C for 2 days. The resulting crystals were washed with DMF (3 *×* 5 mL) and dried in air. Yield 0.0326 g (64 %). Crystal Data for C_62_H_56_N_8_O_16_S_6_Co_3_ (*M* = 1528.29 g/mol): monoclinic, space group *P*2_1_/*c* (no. 14), *a* = 10.852(2) Å, *b* = 25.406(5) Å, *c* = 12.801(2) Å, β = 109.74(3)°, *V* = 3322.0(12) Å^3^, *Z* = 2, *T* = 100 K, μ(0.793127 Å) = 1.345 mm^−1^, *D*_calc_ = 1.538 g⋅cm^−3^, 19683 reflections measured (4.80° ≤ 2θ ≤ 61.96°), 7317 unique (*R*_int_ = 0.0514) which were used in all calculations. The final *R*_1_ was 0.0506 (*I* > 2 σ(*I*)), *wR*_2_ was 0.1429 (all data) and GoF = 1.052. Anal. Calc. for [Co_3_(btdc)_3_(bpy)_2_]∙3DMF∙2H_2_O = C_59_H_53_N_7_O_17_S_6_Co_3_ (%): C 47.2, H 3.6, N 6.5, S 12.8 %. Found: C 47.2, H 3.4, N 6.5, S 12.6 %. IR data (cm^−1^): 429(w), 654(w), 733(w), 768(m), 798(w), 825(w), 1045(w), 1089(w), 1376(s), 1440(s), 1477(m), 1517(m), 1544(m), 1559(m), 1598(s), 1665(m), 2856(w), 2925(w), 2962(w), 3090(w), 3428(w, broad).

Synthesis of [Co_3_(btdc)_3_(pz)(dmf)_2_]∙4DMF∙1.5H_2_O (**2**). CAUTION: the perchloric acid and its salts are potentially explosive! Cobalt(II) perchlorate hexahydrate (36.6 mg, 0.1 mmol), 2,2′-bithiophen-5,5′-dicarboxylic acid (H_2_btdc, 25.4 mg, 0.1 mmol), pyrazine (pz, 7.0 mg, 0.088 mmol), 10 μL of concentrated HClO_4_ and 5 mL of *N*,*N*-dimethylformamide (DMF) were placed in a glass vial with a screw cap. The reaction mixture was sonicated for 30 min, and then heated at 105 °C for 2 days. The resulting crystals were washed with DMF (3 *×* 5 mL) and dried in air. Yield 0.0336 g (68 %). Crystal Data for C_52_H_61_N_8_O_19.5_S_6_Co_3_ (*M* = 1479.23 g/mol): orthorhombic, space group *Fddd* (no. 70), *a* = 11.746(2) Å, *b* = 42.150(4) Å, *c* = 51.849(2) Å, *V* = 25670(5) Å^3^, *Z* = 16, *T* = 100 K, μ(0.793127 Å) = 1.392 mm^−1^, *D*_calc_ = 1.531 g⋅cm^−3^, 50295 reflections measured (2.78° ≤ 2θ ≤ 62.06°), 7189 unique (*R*_int_ = 0.0585) which were used in all calculations. The final *R*_1_ was 0.0361 (*I* > 2σ(*I*)), *wR*_2_ was 0.0997 (all data) and GoF = 1.073. Anal. Calc. for [Co_3_(btdc)_3_(pz)(dmf)_2_]∙3.7DMF∙5.8H_2_O = C_51,1_H_67,5_N_7,7_O_23.5_S_6_Co_3_(%): C 40.0, H 4.4, N 7.0, S 12.5 %. Found: C 40.2, H 4.0, N 6.7, S 12.0 %. IR data (cm^−1^): 419(w), 443(w), 468(w), 650(w), 690(w), 771(m), 800(w), 823(w), 884(w), 1039(w), 1112(w), 1385(s), 1436(s), 1517(m), 1589(m), 1654(s), 2854(m), 2926(m), 3449(w, broad).

Synthesis of [Co_3_(btdc)_3_(dmf)_4_]∙2DMF∙2H_2_O (**3**). Cobalt(II) nitrate hexahydrate (29.7 mg, 0.1 mmol), 2,2′-bithiophen-5,5′-dicarboxylic acid (H_2_btdc, 25.4 mg, 0.1 mmol) and 5 mL of *N*,*N*-dimethylformamide (DMF) were placed in a glass vial with a screw cap. The reaction mixture was sonicated for 30 min, and then heated at 110 °C. Purple block crystals of **3** were isolated after 2 days. Crystal Data for C_48_H_58_N_6_O_20_S_6_Co_3_ (*M* = 1408.15 g/mol): monoclinic, space group *P*2_1_/*c* (no. 14), *a* = 11.96(2) Å, *b* = 25.867(8) Å, *c* = 11.461(16) Å, β = 106.49(3)°, *V* = 3400(8) Å^3^, *Z* = 2, *T* = 100 K, μ(0.793127 Å) = 1.309 mm^−1^, *D*_calc_ = 1.376 g⋅cm^−3^, 28,176 reflections measured (5.16° ≤ 2θ ≤ 64.82°), 7481 unique (*R*_int_ = 0.0782) which were used in all calculations. The final *R*_1_ was 0.0764 (*I* > 2σ(*I*)), *wR*_2_ was 0.2365 (all data) and GoF = 1.044.

## 4. Conclusions

Three new 3D metal-organic frameworks based on Co(II) and 2,2′-bithiophen-5,5′-dicarboxylate (btdc^2–^) Co_3_(btdc)_3_(bpy)_2_]·4DMF, **1**; [Co_3_(btdc)_3_(pz)(dmf)_2_]·4DMF·1.5H_2_O, **2**; [Co_3_(btdc)_3_(dmf)_4_]∙2DMF∙2H_2_O, **3** (bpy = 2,2′-bipyridyl, pz = pyrazine, dmf = *N*,*N*-dimethylformamide) were synthesized and characterized by a number of physical-chemical methods such as powder X-ray diffraction, TG analyses, IR spectroscopy, gas adsorption and magnetic measurements. All title MOFs share the same trinuclear carboxylate building blocks {Co_3_(RCOO)_6_}, connected in three dimensions either by btdc^2–^ ligands (**1**, **3**) or by both btdc^2–^ and pz bridging ligands (**2**). The metal-organic frameworks in **1** and **3** belong to primitive cubic topology while **2** features complex 6-connected network with point symbol 4^10^.6^5^. The permanent porosity of the thermally-activated framework **1** was confirmed by N_2_, CO_2_, CO, O_2_ and CH_4_ adsorption isotherm measurements at various temperatures. Most interestingly, porous material **1** combines substantial CO_2_/N_2_, CO_2_/CO and CO_2_/O_2_ separation selectivities as well as low CO_2_ adsorption heat which provide a promising opportunity for the future development of an efficient gas separation technology. The study of magnetic properties of compounds **1** and **2** revealed their mainly paramagnetic nature with weak antiferromagnetic interactions between the Co(II) cations within the {Co_3_(RCOO)_6_} units. Moreover, the shorter pz linkers in **2** reduce the spacing between those carboxylate units, contrary to **1**, where such moieties are solely separated by longer btdc^2–^ ligands. This results in an emergence of additional antiferromagnetic interactions between the trinuclear units in **2** at *T* < 20 K.

## Figures and Tables

**Table 1:** Gas uptakes on **1** at 273 K and 298 K and 1 bar.

Gas	273 K			298 K		
	mL(STP)/g	mmol/g	wt. %	mL(STP)/g	mmol/g	wt. %
CO_2_	57.2	2.55	11.2	35.7	1.59	7.0
CH_4_	22.5	1.00	1.6	13.4	0.60	1.0
N_2_	4.7	0.21	0.6	3.5	0.16	0.4
O_2_	3.5	0.16	0.5	2.4	0.11	0.3
CO	6.9	0.31	0.9	3.9	0.17	0.5

**Table 2:** Selectivity factors for separation of binary gas mixtures evaluated by different approaches.

273 K				298 K			
CO_2_/N_2_	CO_2_/CH_4_	CO_2_/O_2_	CO_2_/CO	CO_2_/N_2_	CO_2_/CH_4_	CO_2_/O_2_	CO_2_/CO
**Selectivity factors as ratio of adsorbed amount *S* = *V*_1_/*V*_2_**							
12.2	2.5	16.3	8.3	10.2	2.7	14.9	9.2
**Selectivity factors as ratio of Henry constants *S* = *K_H_*_1_/*K_H_*_2_**							
35.7	4.8	45.4	20.8	18.9	3.8	25.4	15.1
**IAST selectivity at total pressure 1 bar and gas mixture composition 1:1**							
10.0 16.3	3.5	11.5	7.9	7.9 12.8	3.3	9.4	7.2

^a^ CO_2_:N_2_ = 15:85 (*v*/*v*).

## Data Availability

Date of the compounds are available from the authors.

## Supplementary Materials

The following are available online. Table S1: Crystal data and structure refinement for **1**–**3**; Figure S1: Coordination environment of Co(II) cations in **1** (a) and **3** (b). (a) Ellipsoids of 50% probability. Symmetry code: (i): 1 − x, 1 − y, 1 – z; (ii) x, ½ − y, ½ + z; (iii) 1 – x, ½ + y, ½ − z. (b) Ellipsoids of 30% probability. Symmetry code: (i): 1 − x, 1 − y, 1 – z; (ii) 1 − x, ½ + y, 3/2 − z; (iii) x, ½ − y, z − ½. Only one of the possible orientations of coordinated DMF molecules and btdc^2−^ ligands is shown. Hydrogen atoms are not shown; Figure S2: Coordination environment of Co(II) cations in **2**. Ellipsoids of 50% probability. Hydrogen atoms are not shown. Symmetry code (i): 1/2 − x, 1 − y, 3/2 − z; Figure S3: Powder X-ray diffraction patterns for compounds **1** and **2** (experimentally observed—in blue and red, respectively, theoretical—in light-blue and pink, respectively); Figure S4: The IR spectrum of the compound **1**; Figure S5: The IR spectrum of the compound **2**; Table S2: Virial coefficients *A_i_* and *B_i_* for gas adsorption isotherms at 273 K and 298 K on **1**; Figure S6: Semilogarithmic representation of nitrogen adsorption (filled squares) and desorption (open squares) isotherms at 77 K for the compound **1**; Figure S7: Nitrogen adsorption isotherm fit (from pore size distrubution calculation); Figure S8: Pore size distributions for **1** calculated by DFT (N_2_ adsorption at 77 K) and Zeo++ [1,2]; Figure S9: Fits of isotherms by virial equations; Figure S10: Isosteric heats of gas adsorption on **1**; Table S3: Henry constants for gas adsorption on **1** in mmol·g^−1^·bar^−1^ at 273 K and 298 K; Table S4: Fitted parameters for adsorption isotherms on **1** at 273 K and 298 K; Figure S11: Fits of the gas adsorption isotherms by appropriate models; Figure S12: Prediction of adsorption equilibrium by IAST (solid lines) and dependence of selectivity factors on gas phase composition (dashed lines) for binary gas mixtures: (a) CO_2_/N_2_; (b) CO_2_/CH_4_; (c) CO_2_/O_2_; (d) CO_2_/CO; Figure S13: Temperature dependences of the magnetic susceptibility for compounds **1** (left) and **2** (right). The magnetic susceptibility of both compounds exhibits dependence neither on magneto-thermal history, nor on the magnetic field value, except for the lowest temperatures where the curves measured at 1 and 10 kOe diverge slightly due to nonlinear M(H) dependence.
