# Supplementary material for: 3D Metal–Organic Frameworks Based on Co(II) and Bithiophendicarboxylate: Synthesis, Crystal Structures, Gas Adsorption, and Magnetic Properties
Source: Molecules. 2021 Feb 26;26(5):1269. doi: 10.3390/molecules26051269 (PMC7956319; doi:10.3390/molecules26051269)

checkCIF/PLATON report

Structure factors have been supplied for datablock(s) ssk-1b

THIS REPORT IS FOR GUIDANCE ONLY. IF USED AS PART OF A REVIEW PROCEDURE FOR PUBLICATION, IT SHOULD NOT REPLACE THE EXPERTISE OF AN EXPERIENCED CRYSTALLOGRAPHIC REFEREE.

No syntax errors found. CIF dictionary Interpreting this report

Datablock: ssk-1b

```
Bond precision:   C-C = 0.0048 A          Wavelength=0.79313

Cell:             a=10.852(2)     b=25.406(5)        c=12.801(2)
                  alpha=90        beta=109.74(3)     gamma=90
Temperature:      100 K
```

	Calculated	Reported
Volume	3321.9(12)	3322.0(12)
Space group	P 21/c	P 21/c
Hall group	-P 2ybc	-P 2ybc
Moiety formula	C50 H28 Co3 N4 O12 S6, 4(C3 H7 N O)	?
Sum formula	C62 H56 Co3 N8 O16 S6	C62 H56 Co3 N8 O16 S6
Mr	1538.30	1538.29
Dx,g cm-3	1.538	1.538
Z	2	2
Mu (mm-1)	1.344	1.345
F000	1578.0	1578.0
F000’	1582.70	
h,k,lmax	14,32,16	13,32,16
Nref	7601	7317
Tmin,Tmax	0.817,0.935	0.893,1.000
Tmin’	0.817	

```
Correction method= # Reported T Limits: Tmin=0.893 Tmax=1.000
AbsCorr = MULTI-SCAN

Data completeness= 0.963          Theta(max)= 30.977

R(reflections)= 0.0506( 6412)     wR2(reflections)= 0.1429( 7317)

S = 1.052                  Npar= 530
```

The following ALERTS were generated. Each ALERT has the format
test-name_ALERT_alert-type_alert-level.
Click on the hyperlinks for more details of the test.

Alert level C

```
PLAT029_ALERT_3_C _diffrn_measured_fraction_theta_full value Low .      0.970 Why?
PLAT220_ALERT_2_C NonSolvent Resd 1  C   Ueq(max) / Ueq(min) Range        3.6 Ratio
PLAT911_ALERT_3_C Missing FCF Refl Between Thmin & STh/L=    0.600        170 Report
PLAT913_ALERT_3_C Missing # of Very Strong Reflections in FCF ....          7 Note
```

Alert level G

```
ABSMU01_ALERT_1_G  Calculation of _exptl_absorpt_correction_mu
                not performed for this radiation type.
PLAT002_ALERT_2_G Number of Distance or Angle Restraints on AtSite         20 Note
PLAT003_ALERT_2_G Number of Uiso or Uij Restrained non-H Atoms ...         32 Report
PLAT004_ALERT_5_G Polymeric Structure Found with Maximum Dimension          3 Info
PLAT092_ALERT_4_G Check: Wavelength Given is not Cu,Ga,Mo,Ag,In Ka    0.79313 Ang.
PLAT176_ALERT_4_G The CIF-Embedded .res File Contains SADI Records          3 Report
PLAT178_ALERT_4_G The CIF-Embedded .res File Contains SIMU Records          2 Report
PLAT302_ALERT_4_G Anion/Solvent/Minor-Residue Disorder (Resd  2  )       100% Note
PLAT302_ALERT_4_G Anion/Solvent/Minor-Residue Disorder (Resd  3  )       100% Note
PLAT302_ALERT_4_G Anion/Solvent/Minor-Residue Disorder (Resd  4  )       100% Note
PLAT302_ALERT_4_G Anion/Solvent/Minor-Residue Disorder (Resd  5  )       100% Note
PLAT794_ALERT_5_G Tentative Bond Valency for Co1       (II)      .       2.06 Info
PLAT794_ALERT_5_G Tentative Bond Valency for Co2       (II)      .       2.04 Info
PLAT860_ALERT_3_G Number of Least-Squares Restraints .............        334 Note
PLAT910_ALERT_3_G Missing # of FCF Reflection(s) Below Theta(Min).          3 Note
PLAT912_ALERT_4_G Missing # of FCF Reflections Above STh/L=  0.600        100 Note
PLAT933_ALERT_2_G Number of OMIT Records in Embedded .res File ...          7 Note
PLAT961_ALERT_5_G Dataset Contains no Negative Intensities .......     Please Check
PLAT978_ALERT_2_G Number C-C Bonds with Positive Residual Density.          1 Info
PLAT984_ALERT_1_G The  S-f'=    0.1503 Deviates from the B&C-Value     0.1482 Check
PLAT985_ALERT_1_G The Co-f"=    1.1930 Deviates from the B&C-Value     1.1884 Check
PLAT985_ALERT_1_G The  S-f"=    0.1565 Deviates from the B&C-Value     0.1550 Check
PLAT992_ALERT_5_G Repd & Actual _reflns_number_gt Values Differ by          1 Check
```

---

0 **ALERT level A** = Most likely a serious problem - resolve or explain
0 **ALERT level B** = A potentially serious problem, consider carefully
4 **ALERT level C** = Check. Ensure it is not caused by an omission or oversight
23 **ALERT level G** = General information/check it is not something unexpected

4 ALERT type 1 CIF construction/syntax error, inconsistent or missing data
5 ALERT type 2 Indicator that the structure model may be wrong or deficient
5 ALERT type 3 Indicator that the structure quality may be low
8 ALERT type 4 Improvement, methodology, query or suggestion
5 ALERT type 5 Informative message, check

It is advisable to attempt to resolve as many as possible of the alerts in all categories. Often the minor alerts point to easily fixed oversights, errors and omissions in your CIF or refinement strategy, so attention to these fine details can be worthwhile. In order to resolve some of the more serious problems it may be necessary to carry out additional measurements or structure refinements. However, the purpose of your study may justify the reported deviations and the more serious of these should normally be commented upon in the discussion or experimental section of a paper or in the "special_details" fields of the CIF. checkCIF was carefully designed to identify outliers and unusual parameters, but every test has its limitations and alerts that are not important in a particular case may appear. Conversely, the absence of alerts does not guarantee there are no aspects of the results needing attention. It is up to the individual to critically assess their own results and, if necessary, seek expert advice.

Publication of your CIF in IUCr journals

A basic structural check has been run on your CIF. These basic checks will be run on all CIFs submitted for publication in IUCr journals (*Acta Crystallographica*, *Journal of Applied Crystallography*, *Journal of Synchrotron Radiation*); however, if you intend to submit to *Acta Crystallographica Section C* or *E* or *IUCrData*, you should make sure that full publication checks are run on the final version of your CIF prior to submission.

Publication of your CIF in other journals

Please refer to the *Notes for Authors* of the relevant journal for any special instructions relating to CIF submission.

PLATON version of 22/12/2019; check.def file version of 13/12/2019

Datablock ssk-1b - ellipsoid plot

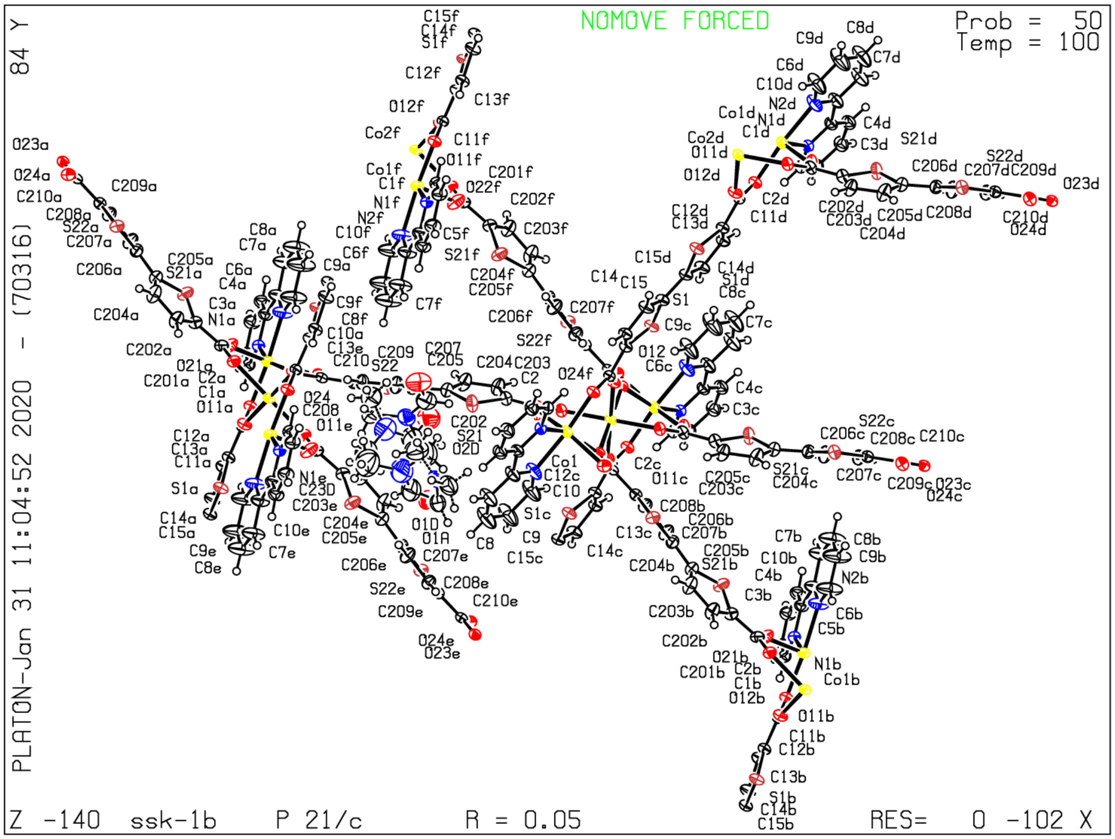

Supplement: Supplementary file 1 [file molecules-26-01269-s001.zip › 1.pdf]
