# Supplementary material for: 3D Metal–Organic Frameworks Based on Co(II) and Bithiophendicarboxylate: Synthesis, Crystal Structures, Gas Adsorption, and Magnetic Properties
Source: Molecules. 2021 Feb 26;26(5):1269. doi: 10.3390/molecules26051269 (PMC7956319; doi:10.3390/molecules26051269)

checkCIF/PLATON report

Structure factors have been supplied for datablock(s) pz-8_sq

THIS REPORT IS FOR GUIDANCE ONLY. IF USED AS PART OF A REVIEW PROCEDURE FOR PUBLICATION, IT SHOULD NOT REPLACE THE EXPERTISE OF AN EXPERIENCED CRYSTALLOGRAPHIC REFEREE.

No syntax errors found. CIF dictionary Interpreting this report

Datablock: pz-8_sq

Bond precision: C-C = 0.0030 A Wavelength=0.79313

Cell:	a=11.746(2)	b=42.150(4)	c=51.849(2)
	alpha=90	beta=90	gamma=90
Temperature:	100 K		

	Calculated	Reported
Volume	25670(5)	25670(5)
Space group	F d d d	F d d d
Hall group	-F 2uv 2vw	-F 2uv 2vw
Moiety formula	C40 H30 Co3 N4 O14 S6 [+ solvent]	?
Sum formula	C40 H30 Co3 N4 O14 S6 [+ solvent]	C52 H61 Co3 N8 O19.50 S6
Mr	1159.83	1479.23
Dx,g cm-3	1.200	1.531
Z	16	16
Mu (mm-1)	1.359	1.392
F000	9392.0	12192.0
F000'	9427.86	
h,k,lmax	15,54,67	15,54,64
Nref	7392	7189
Tmin,Tmax	0.920,0.933	0.785,1.000
Tmin'	0.870	

Correction method= # Reported T Limits: Tmin=0.785 Tmax=1.000
AbsCorr = MULTI-SCAN

Data completeness= 0.973 Theta(max)= 31.034

R(reflections)= 0.0361(6073) wR2(reflections)= 0.0997(7189)

S = 1.073 Npar= 307

The following ALERTS were generated. Each ALERT has the format
test-name_ALERT_alert-type_alert-level.
Click on the hyperlinks for more details of the test.

Alert level C

```
PLAT220_ALERT_2_C NonSolvent Resd 1  C   Ueq(max) / Ueq(min) Range        3.5 Ratio
PLAT242_ALERT_2_C Low    MainMol Ueq as Compared to Neighbors of       N1D Check
PLAT911_ALERT_3_C Missing FCF Refl Between Thmin & STh/L=    0.600        111 Report
PLAT913_ALERT_3_C Missing # of Very Strong Reflections in FCF ....          6 Note
```

Alert level G

```
FORMU01_ALERT_2_G  There is a discrepancy between the atom counts in the
            _chemical_formula_sum and the formula from the _atom_site* data.
            Atom count from _chemical_formula_sum:C52 H61 Co3 N8 O19.5 S6
            Atom count from the _atom_site data:  C40 H30 Co3 N4 O14 S6
ABSMU01_ALERT_1_G  Calculation of _exptl_absorpt_correction_mu
                not performed for this radiation type.
CELLZ01_ALERT_1_G Difference between formula and atom_site contents detected.
CELLZ01_ALERT_1_G ALERT: Large difference may be due to a
            symmetry error - see SYMMG tests
           From the CIF: _cell_formula_units_Z   16
           From the CIF: _chemical_formula_sum  C52 H61 Co3 N8 O19.50 S6
           TEST: Compare cell contents of formula and atom_site data

           atom    Z*formula  cif sites diff
           C        832.00    640.00  192.00
           H        976.00    480.00  496.00
           Co        48.00     48.00    0.00
           N        128.00     64.00   64.00
           O        312.00    224.00   88.00
           S         96.00     96.00    0.00
PLAT003_ALERT_2_G Number of Uiso or Uij Restrained non-H Atoms ...          1 Report
PLAT041_ALERT_1_G Calc. and Reported SumFormula    Strings  Differ     Please Check
PLAT051_ALERT_1_G Mu(calc) and Mu(CIF) Ratio Differs from 1.0 by .       2.34 %
PLAT068_ALERT_1_G Reported F000 Differs from Calcd (or Missing)...     Please Check
PLAT083_ALERT_2_G SHELXL Second Parameter in WGHT  Unusually Large      38.46 Why ?
PLAT092_ALERT_4_G Check: Wavelength Given is not Cu,Ga,Mo,Ag,In Ka    0.79313 Ang.
PLAT186_ALERT_4_G The CIF-Embedded .res File Contains ISOR Records          1 Report
PLAT606_ALERT_4_G VERY LARGE Solvent Accessible VOID(S) in Structure        ! Info
PLAT794_ALERT_5_G Tentative Bond Valency for Co1       (II)      .       2.04 Info
PLAT794_ALERT_5_G Tentative Bond Valency for Co2       (II)      .       1.99 Info
PLAT804_ALERT_5_G Number of ARU-Code Packing Problem(s) in PLATON          45 Info
PLAT860_ALERT_3_G Number of Least-Squares Restraints .............          6 Note
PLAT869_ALERT_4_G ALERTS Related to the Use of SQUEEZE Suppressed           ! Info
PLAT912_ALERT_4_G Missing # of FCF Reflections Above STh/L=  0.600         70 Note
PLAT952_ALERT_5_G Calculated (ThMax) and CIF-Reported Lmax Differ           3 Units
PLAT958_ALERT_1_G Calculated (ThMax) and Actual (FCF) Lmax Differ           3 Units
PLAT961_ALERT_5_G Dataset Contains no Negative Intensities .......     Please Check
PLAT978_ALERT_2_G Number C-C Bonds with Positive Residual Density.          2 Info
PLAT984_ALERT_1_G The  S-f'=    0.1503 Deviates from the B&C-Value     0.1482 Check
PLAT985_ALERT_1_G The Co-f"=    1.1930 Deviates from the B&C-Value     1.1884 Check
PLAT985_ALERT_1_G The  S-f"=    0.1565 Deviates from the B&C-Value     0.1550 Check
```

---

0 **ALERT level A** = Most likely a serious problem - resolve or explain
0 **ALERT level B** = A potentially serious problem, consider carefully
4 **ALERT level C** = Check. Ensure it is not caused by an omission or oversight
25 **ALERT level G** = General information/check it is not something unexpected

```
10 ALERT type 1 CIF construction/syntax error, inconsistent or missing data
 6 ALERT type 2 Indicator that the structure model may be wrong or deficient
 3 ALERT type 3 Indicator that the structure quality may be low
 5 ALERT type 4 Improvement, methodology, query or suggestion
 5 ALERT type 5 Informative message, check
```

It is advisable to attempt to resolve as many as possible of the alerts in all categories. Often the minor alerts point to easily fixed oversights, errors and omissions in your CIF or refinement strategy, so attention to these fine details can be worthwhile. In order to resolve some of the more serious problems it may be necessary to carry out additional measurements or structure refinements. However, the purpose of your study may justify the reported deviations and the more serious of these should normally be commented upon in the discussion or experimental section of a paper or in the "special_details" fields of the CIF. checkCIF was carefully designed to identify outliers and unusual parameters, but every test has its limitations and alerts that are not important in a particular case may appear. Conversely, the absence of alerts does not guarantee there are no aspects of the results needing attention. It is up to the individual to critically assess their own results and, if necessary, seek expert advice.

Publication of your CIF in IUCr journals

A basic structural check has been run on your CIF. These basic checks will be run on all CIFs submitted for publication in IUCr journals (*Acta Crystallographica*, *Journal of Applied Crystallography*, *Journal of Synchrotron Radiation*); however, if you intend to submit to *Acta Crystallographica Section C* or *E* or *IUCrData*, you should make sure that full publication checks are run on the final version of your CIF prior to submission.

Publication of your CIF in other journals

Please refer to the *Notes for Authors* of the relevant journal for any special instructions relating to CIF submission.

PLATON version of 22/12/2019; check.def file version of 13/12/2019

Datablock pz-8_sq - ellipsoid plot

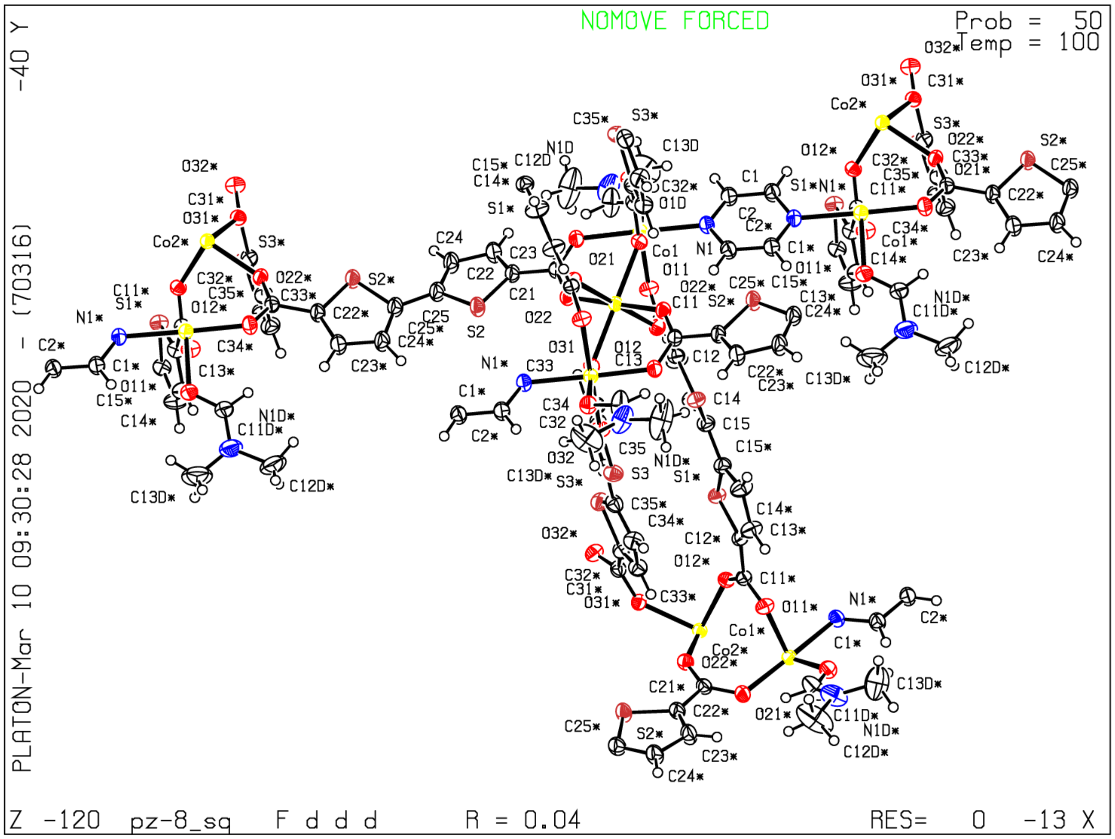

Supplement: Supplementary file 1 [file molecules-26-01269-s001.zip › 2.pdf]
