# Supplementary material for: 3D Metal–Organic Frameworks Based on Co(II) and Bithiophendicarboxylate: Synthesis, Crystal Structures, Gas Adsorption, and Magnetic Properties
Source: Molecules. 2021 Feb 26;26(5):1269. doi: 10.3390/molecules26051269 (PMC7956319; doi:10.3390/molecules26051269)

checkCIF/PLATON report

Structure factors have been supplied for datablock(s) pz-2_sq

THIS REPORT IS FOR GUIDANCE ONLY. IF USED AS PART OF A REVIEW PROCEDURE FOR PUBLICATION, IT SHOULD NOT REPLACE THE EXPERTISE OF AN EXPERIENCED CRYSTALLOGRAPHIC REFEREE.

No syntax errors found. CIF dictionary Interpreting this report

Datablock: pz-2_sq

Bond precision:	C-C = 0.0087 A		Wavelength=0.79313
Cell:	a=11.96(2)	b=25.867(8)	c=11.461(16)
	alpha=90	beta=106.49(3)	gamma=90
Temperature:	100 K		

	Calculated	Reported
Volume	3400(7)	3400(8)
Space group	P 21/c	P 21/c
Hall group	-P 2ybc	-P 2ybc
Moiety formula	C42 H40 Co3 N4 O16 S6 [+ solvent]	?
Sum formula	C42 H40 Co3 N4 O16 S6 [+ solvent]	C48 H58 Co3 N6 O20 S6
Mr	1225.93	1408.15
Dx,g cm-3	1.198	1.376
Z	2	2
Mu (mm-1)	1.290	1.309
F000	1250.0	1450.0
F000’	1254.55	
h,k,lmax	16,34,15	14,33,15
Nref	8800	7481
Tmin,Tmax	0.937,0.974	0.716,1.000
Tmin’	0.937	

Correction method= # Reported T Limits: Tmin=0.716 Tmax=1.000
AbsCorr = MULTI-SCAN

Data completeness= 0.850 Theta(max)= 32.413

R(reflections)= 0.0762(4702) wR2(reflections)= 0.2365(7481)

S = 1.044 Npar= 401

The following ALERTS were generated. Each ALERT has the format
test-name_ALERT_alert-type_alert-level.
Click on the hyperlinks for more details of the test.

Alert level C

```
PLAT029_ALERT_3_C _diffrn_measured_fraction_theta_full value Low .      0.960 Why?
PLAT148_ALERT_3_C s.u. on the      a    - Axis is (Too) Large ....      0.020 Ang.
PLAT148_ALERT_3_C s.u. on the      c    - Axis is (Too) Large ....      0.016 Ang.
PLAT220_ALERT_2_C NonSolvent Resd 1  C   Ueq(max) / Ueq(min) Range        3.3 Ratio
PLAT241_ALERT_2_C High   MainMol Ueq as Compared to Neighbors of        O21 Check
PLAT242_ALERT_2_C Low    MainMol Ueq as Compared to Neighbors of        Co1 Check
PLAT341_ALERT_3_C Low Bond Precision on  C-C Bonds ...............    0.00873 Ang.
PLAT906_ALERT_3_C Large K Value in the Analysis of Variance ......      3.976 Check
PLAT911_ALERT_3_C Missing FCF Refl Between Thmin & STh/L=    0.600        239 Report
PLAT913_ALERT_3_C Missing # of Very Strong Reflections in FCF ....          5 Note
PLAT978_ALERT_2_C Number C-C Bonds with Positive Residual Density.          0 Info
```

Alert level G

```
FORMU01_ALERT_2_G  There is a discrepancy between the atom counts in the
            _chemical_formula_sum and the formula from the _atom_site* data.
            Atom count from _chemical_formula_sum:C48 H58 Co3 N6 O20 S6
            Atom count from the _atom_site data:  C42 H40 Co3 N4 O16 S6
ABSMU01_ALERT_1_G  Calculation of _exptl_absorpt_correction_mu
                not performed for this radiation type.
CELLZ01_ALERT_1_G Difference between formula and atom_site contents detected.
CELLZ01_ALERT_1_G ALERT: Large difference may be due to a
            symmetry error - see SYMMG tests
           From the CIF: _cell_formula_units_Z    2
           From the CIF: _chemical_formula_sum  C48 H58 Co3 N6 O20 S6
           TEST: Compare cell contents of formula and atom_site data

           atom    Z*formula  cif sites diff
           C         96.00     84.00   12.00
           H        116.00     80.00   36.00
           Co         6.00      6.00    0.00
           N         12.00      8.00    4.00
           O         40.00     32.00    8.00
           S         12.00     12.00    0.00
PLAT002_ALERT_2_G Number of Distance or Angle Restraints on AtSite         19 Note
PLAT003_ALERT_2_G Number of Uiso or Uij Restrained non-H Atoms ...         23 Report
PLAT004_ALERT_5_G Polymeric Structure Found with Maximum Dimension          3 Info
PLAT041_ALERT_1_G Calc. and Reported SumFormula    Strings  Differ     Please Check
PLAT068_ALERT_1_G Reported F000 Differs from Calcd (or Missing)...     Please Check
PLAT072_ALERT_2_G SHELXL First  Parameter in WGHT  Unusually Large       0.15 Report
PLAT092_ALERT_4_G Check: Wavelength Given is not Cu,Ga,Mo,Ag,In Ka    0.79313 Ang.
PLAT174_ALERT_4_G The CIF-Embedded .res File Contains FLAT Records          4 Report
PLAT176_ALERT_4_G The CIF-Embedded .res File Contains SADI Records          8 Report
PLAT178_ALERT_4_G The CIF-Embedded .res File Contains SIMU Records          3 Report
PLAT186_ALERT_4_G The CIF-Embedded .res File Contains ISOR Records          1 Report
PLAT301_ALERT_3_G Main Residue  Disorder ..............(Resd  1  )        22% Note
PLAT413_ALERT_2_G Short Inter XH3 .. XHn     H22A     ..H203     .       2.11 Ang.
                                                -1+x,y,z  =      1_455 Check
PLAT413_ALERT_2_G Short Inter XH3 .. XHn     H22A     ..H23A     .       2.02 Ang.
                                                -1+x,y,z  =      1_455 Check
PLAT413_ALERT_2_G Short Inter XH3 .. XHn     H22C     ..H23A     .       1.91 Ang.
                                                -1+x,y,z  =      1_455 Check
PLAT432_ALERT_2_G Short Inter X...Y Contact  C13      ..C21D             3.17 Ang.
                                              -x,1-y,1-z  =      3_566 Check
PLAT606_ALERT_4_G VERY LARGE Solvent Accessible VOID(S) in Structure        ! Info
```

```
PLAT794_ALERT_5_G Tentative Bond Valency for Co2       (II)      .       2.02 Info
PLAT860_ALERT_3_G Number of Least-Squares Restraints .............        319 Note
PLAT869_ALERT_4_G ALERTS Related to the Use of SQUEEZE Suppressed            ! Info
PLAT910_ALERT_3_G Missing # of FCF Reflection(s) Below Theta(Min).           4 Note
PLAT912_ALERT_4_G Missing # of FCF Reflections Above STh/L=  0.600         926 Note
PLAT933_ALERT_2_G Number of OMIT Records in Embedded .res File ...           3 Note
PLAT950_ALERT_5_G Calculated (ThMax) and CIF-Reported Hmax Differ            2 Units
PLAT956_ALERT_1_G Calculated (ThMax) and Actual (FCF) Hmax Differ            2 Units
PLAT961_ALERT_5_G Dataset Contains no Negative Intensities .......     Please Check
PLAT984_ALERT_1_G The  S-f'=    0.1503 Deviates from the B&C-Value     0.1482 Check
PLAT985_ALERT_1_G The Co-f"=    1.1930 Deviates from the B&C-Value     1.1884 Check
PLAT985_ALERT_1_G The  S-f"=    0.1565 Deviates from the B&C-Value     0.1550 Check
```

```
   0 ALERT level A = Most likely a serious problem - resolve or explain
   0 ALERT level B = A potentially serious problem, consider carefully
  11 ALERT level C = Check. Ensure it is not caused by an omission or oversight
  33 ALERT level G = General information/check it is not something unexpected

   9 ALERT type 1 CIF construction/syntax error, inconsistent or missing data
  13 ALERT type 2 Indicator that the structure model may be wrong or deficient
  10 ALERT type 3 Indicator that the structure quality may be low
   8 ALERT type 4 Improvement, methodology, query or suggestion
   4 ALERT type 5 Informative message, check
```

It is advisable to attempt to resolve as many as possible of the alerts in all categories. Often the minor alerts point to easily fixed oversights, errors and omissions in your CIF or refinement strategy, so attention to these fine details can be worthwhile. In order to resolve some of the more serious problems it may be necessary to carry out additional measurements or structure refinements. However, the purpose of your study may justify the reported deviations and the more serious of these should normally be commented upon in the discussion or experimental section of a paper or in the "special_details" fields of the CIF. checkCIF was carefully designed to identify outliers and unusual parameters, but every test has its limitations and alerts that are not important in a particular case may appear. Conversely, the absence of alerts does not guarantee there are no aspects of the results needing attention. It is up to the individual to critically assess their own results and, if necessary, seek expert advice.

Publication of your CIF in IUCr journals

A basic structural check has been run on your CIF. These basic checks will be run on all CIFs submitted for publication in IUCr journals (*Acta Crystallographica*, *Journal of Applied Crystallography*, *Journal of Synchrotron Radiation*); however, if you intend to submit to *Acta Crystallographica Section C* or *E* or *IUCrData*, you should make sure that full publication checks are run on the final version of your CIF prior to submission.

Publication of your CIF in other journals

Please refer to the *Notes for Authors* of the relevant journal for any special instructions relating to CIF submission.

PLATON version of 22/12/2019; check.def file version of 13/12/2019

Datablock pz-2_sq - ellipsoid plot

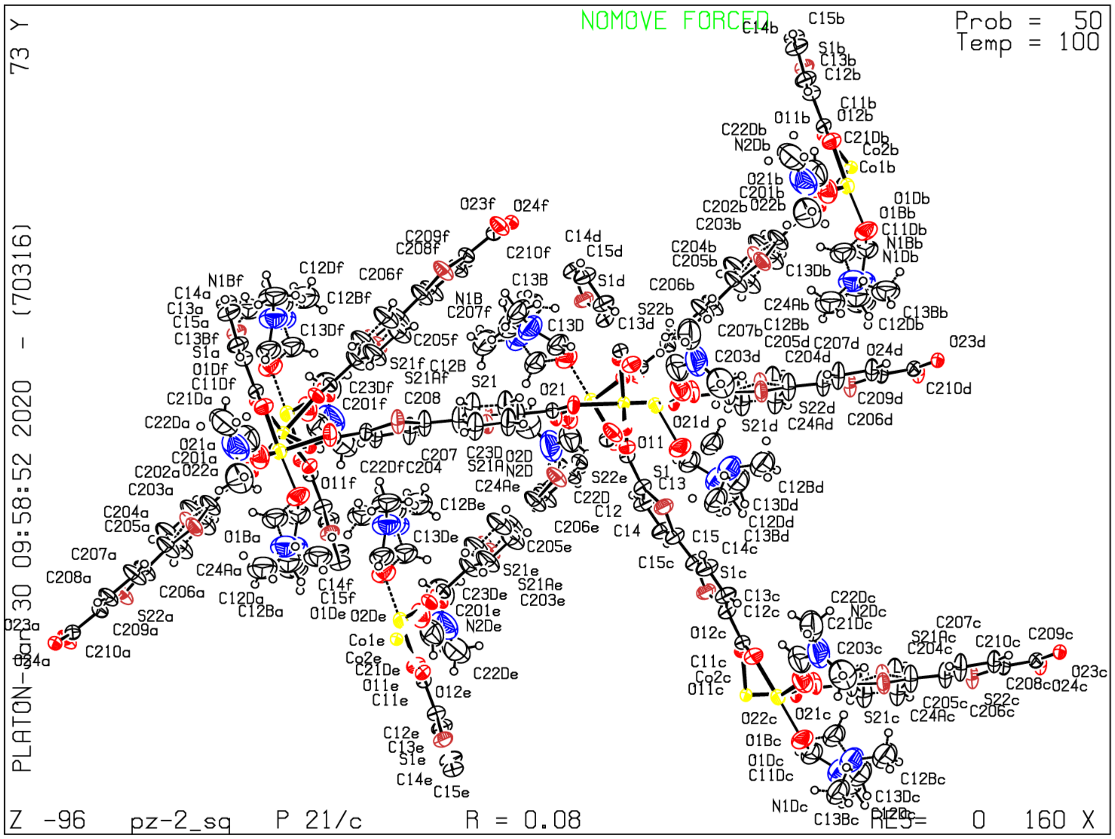

Supplement: Supplementary file 1 [file molecules-26-01269-s001.zip › 3.pdf]
