# Supplementary material for: 3D Metal–Organic Frameworks Based on Co(II) and Bithiophendicarboxylate: Synthesis, Crystal Structures, Gas Adsorption, and Magnetic Properties
Source: Molecules. 2021 Feb 26;26(5):1269. doi: 10.3390/molecules26051269 (PMC7956319; doi:10.3390/molecules26051269)
Supplement: Supplementary file 1 [file molecules-26-01269-s001.zip › SI.pdf]

3D metal-organic frameworks based on Co(II) and bithiophendicarboxylate: synthesis, crystal structures, gas adsorption, and magnetic properties

Vadim A. Dubskikh, Anna A. Lysova, Denis G. Samsonenko, Alexander N. Lavrov, Konstantin A. Kovalenko, Danil N. Dybtsev,* Vladimir P. Fedin

Nikolaev Institute of Inorganic Chemistry, Siberian Branch of the Russian Academy of Sciences, 3 Acad. Lavrentiev Ave., 630090, Novosibirsk, Russia

*Corresponding author, dan@niic.nsc.ru

Table S1. Crystal data and structure refinement for **1–3**.

Identification code	**1**	**2**	**3**
Empirical formula	$C_{62}H_{56}N_8O_{16}S_6Co_3$	$C_{52}H_{61}N_8O_{19.5}S_6Co_3$	$C_{48}H_{58}N_6O_{20}S_6Co_3$
M, g/mol	1528.29	1479.23	1408.15
T, K	100	100	100
Crystal system	*Monoclinic*	*Orthorhombic*	*Monoclinic*
Space group	$P2_1/c$	*Fddd*	$P2_1/c$
a, Å	10.852(2)	11.746(2)	11.96(2)
b, Å	25.406(5)	42.150(4)	25.867(8)
c, Å	12.801(2)	51.849(2)	11.461(16)
α, град.	90	90	90
β, град.	109.74(3)	90	106.49(3)
γ, degree	90	90	90
V, $Å^3$	3322.0(12)	25670(5)	3400(8)
Z	2	16	2
D(calc.), g/cm^3	1.538	1.531	1.376
μ, mm^{-1}	1.345	1.392	1.309
F(000)	1578	12192	1450
Crystal size, mm	0.15 × 0.15 × 0.05	0.10 × 0.05 × 0.05	0.05 × 0.05 × 0.02
θ range for data collection, deg.	2.40–30.98	1.39–31.03	2.58–32.41
Index ranges	$-13 < h < 13$, $-28 < k < 32$, $-16 < l < 16$	$-15 < h < 15$, $-54 < k < 54$, $-59 < l < 64$	$-14 < h < 14$, $-33 < k < 33$, $-15 < l < 15$
Reflections collected / independent	19683 / 7317	50295 / 7189	28176 / 7481
R_{int}	0.0514	0.0585	0.0782
Reflections with $I > 2\sigma(I)$	6412	6073	4702

Goodness-of-fit on F^2	1.052	1.073	1.044
Final R indices [$I > 2\sigma(I)$]	$R_1 = 0.0506$, $wR_2 = 0.1375$	$R_1 = 0.0361$, $wR_2 = 0.0961$	$R_1 = 0.0764$, $wR_2 = 0.2142$
R indices (all data)	$R_1 = 0.0575$, $wR_2 = 0.1429$	$R_1 = 0.0442$ $wR_2 = 0.0997$	$R_1 = 0.1170$, $wR_2 = 0.2365$
Largest diff. peak / hole, e/Å^3	0.723 / −0.738	0.390 / –0.517	0.863 / −0.875

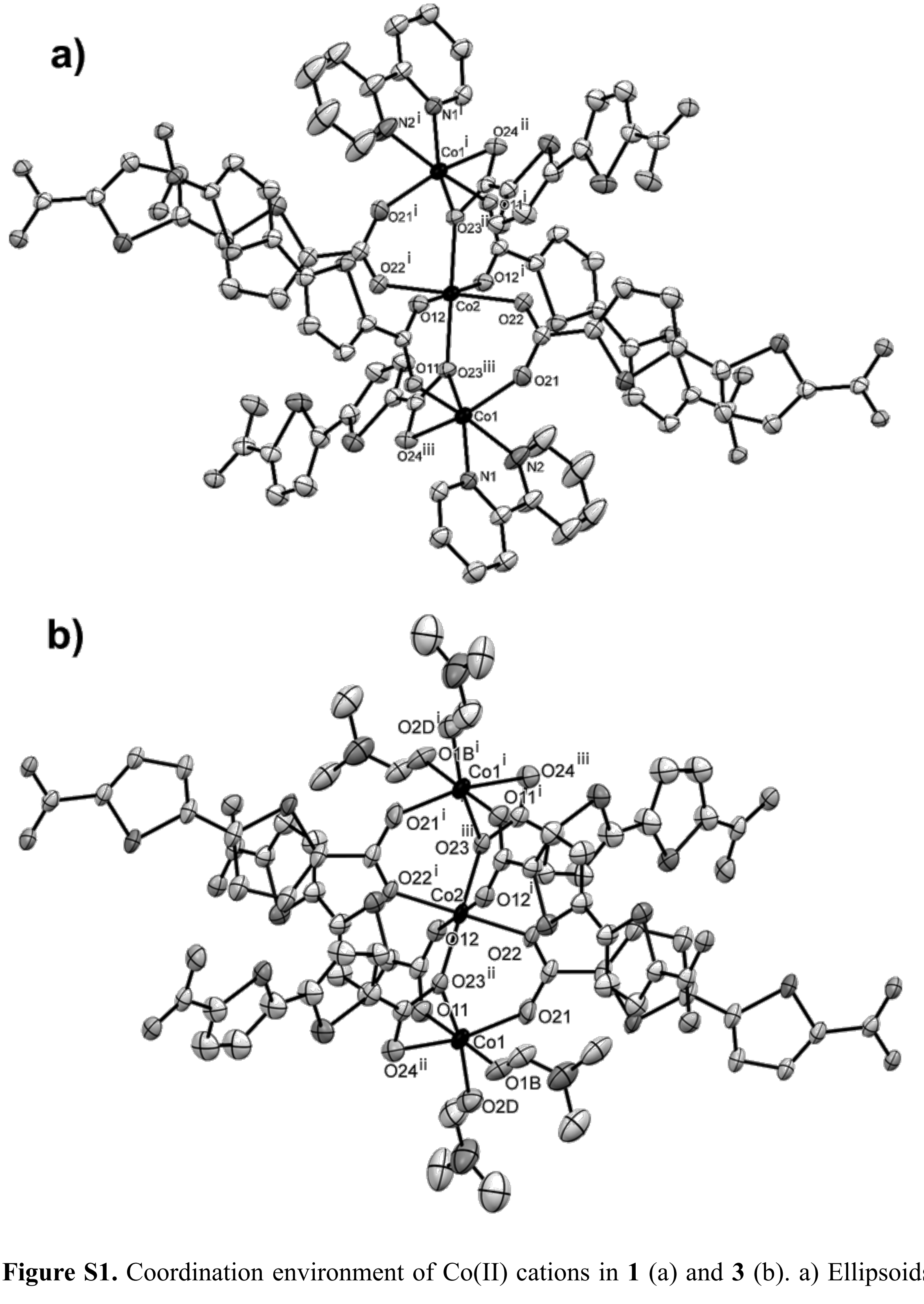

Figure S1. Coordination environment of Co(II) cations in **1** (a) and **3** (b). a) Ellipsoids of 50% probability. Symmetry code: i): 1 - x, 1 - y, 1 – z; ii) x, ½ - y, ½ + z; iii) 1 – x, ½ + y, ½ - z. b) Ellipsoids of 30% probability. Symmetry code: i): 1 - x, 1 - y, 1 – z; ii) 1 - x, ½ + y, 3/2 - z; iii) x, ½ - y, z - ½. Only one of the possible orientations of coordinated DMF molecules and $btdc^{2-}$ ligands is shown. Hydrogen atoms are not shown.

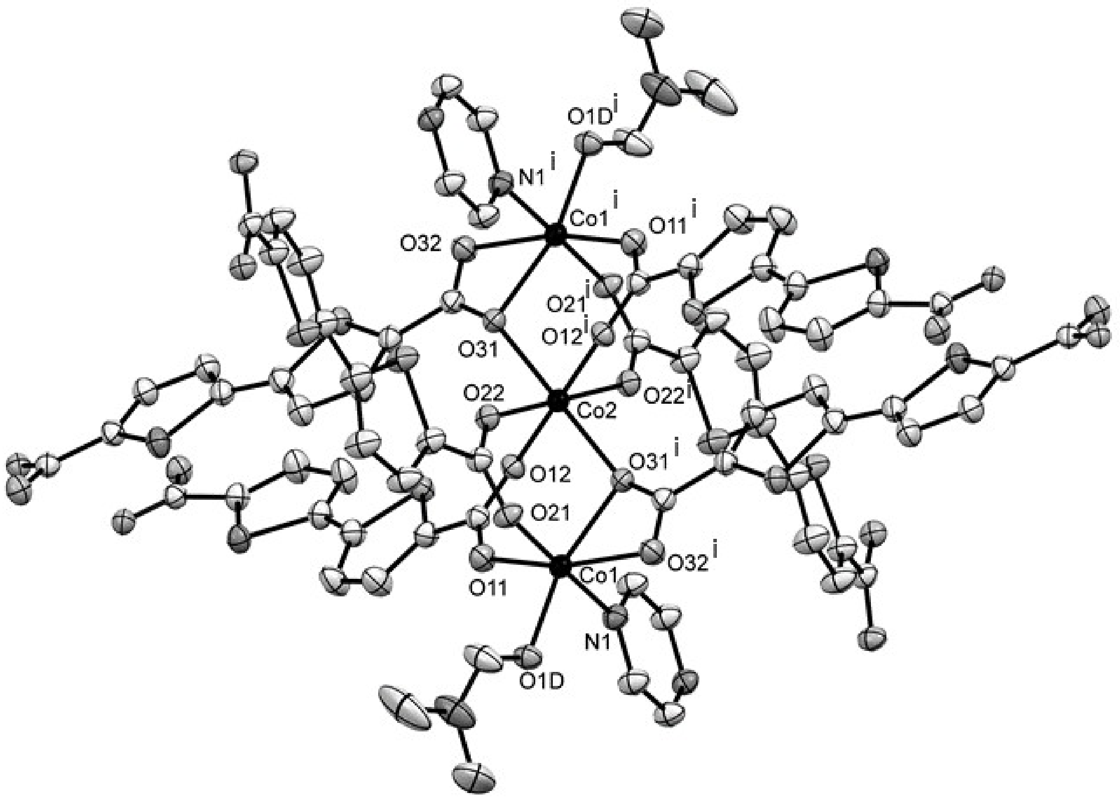

Figure S2. Coordination environment of Co(II) cations in **2**. Ellipsoids of 50% probability. Hydrogen atoms are not shown. Symmetry code (i): 1/2 - x, 1 - y, 3/2 - z.

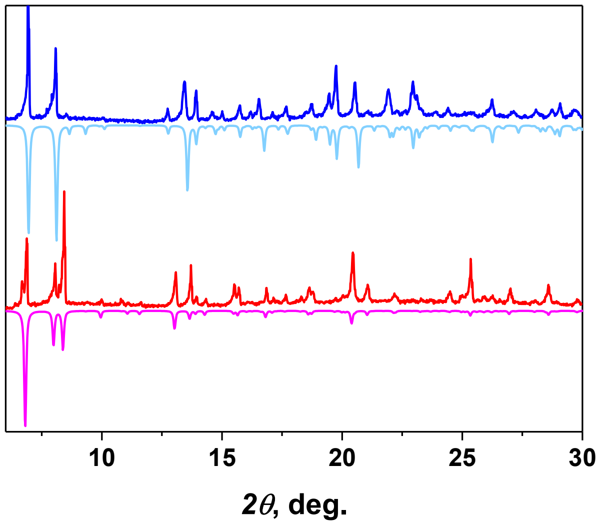

Figure S3. Powder X-ray diffraction patterns for compounds **1** and **2** (experimentally observed – in blue and red, respectively, theoretical – in light-blue and pink, respectively).

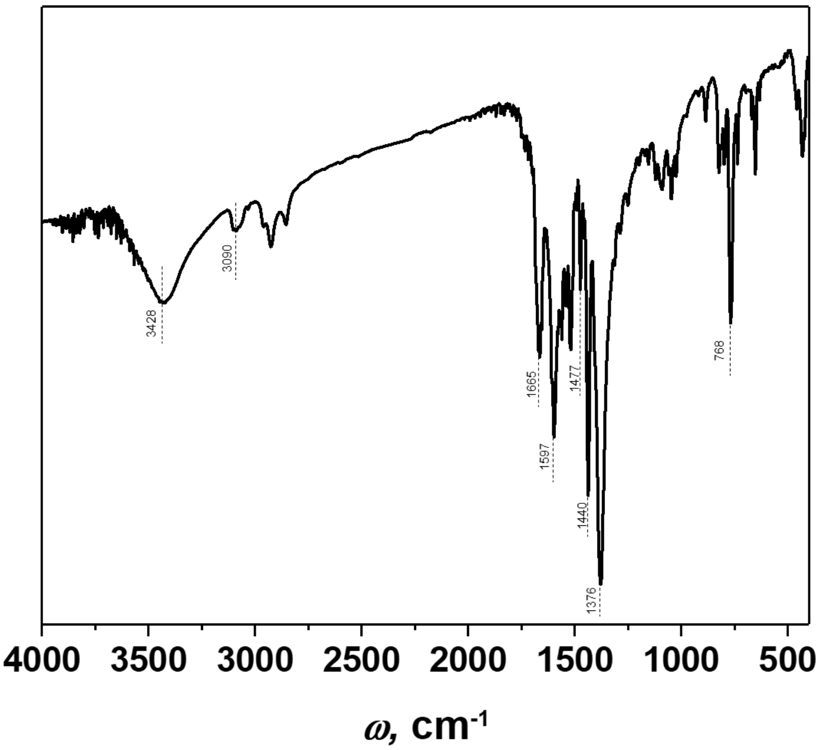

Figure S4. The IR spectrum of the compound **1**.

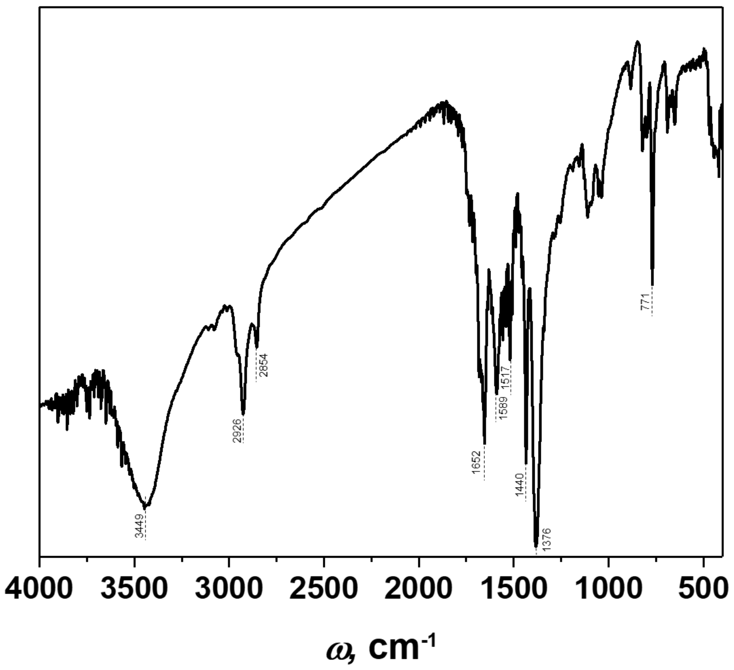

Figure S5. The IR spectrum of the compound **2**.

Isotherms

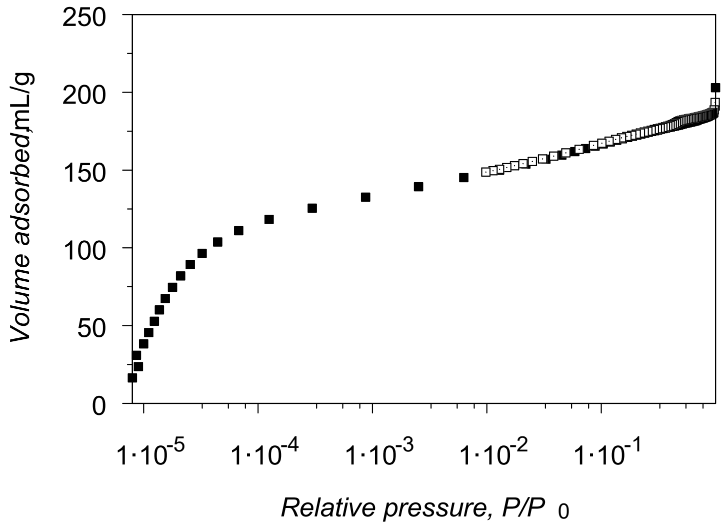

Figure S6. Semilogarithmic representation of nitrogen adsorption (filled squares) and desorption (open squares) isotherms at 77 K for the compound **1**.

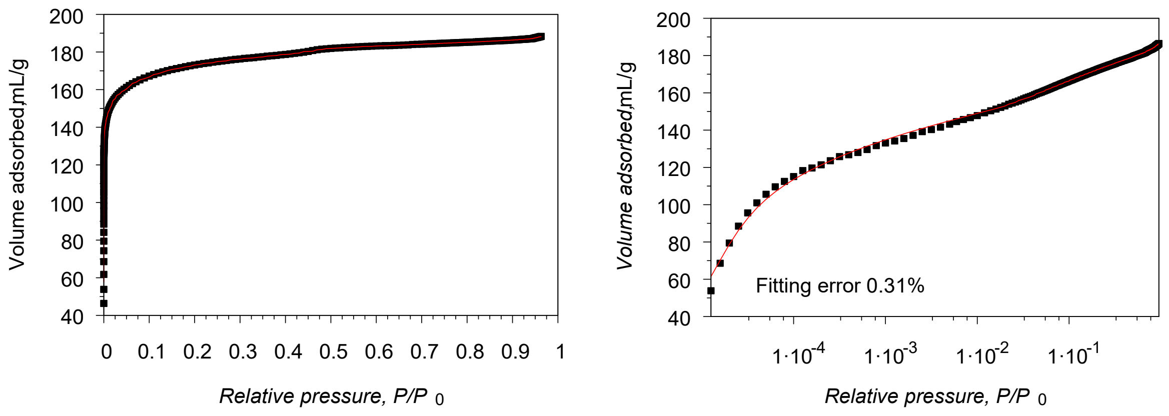

(a) (b)

Figure S7. Nitrogen adsorption isotherm fit (from pore size distribution calculation): a) normal scale; b) semilogarithmic scale.

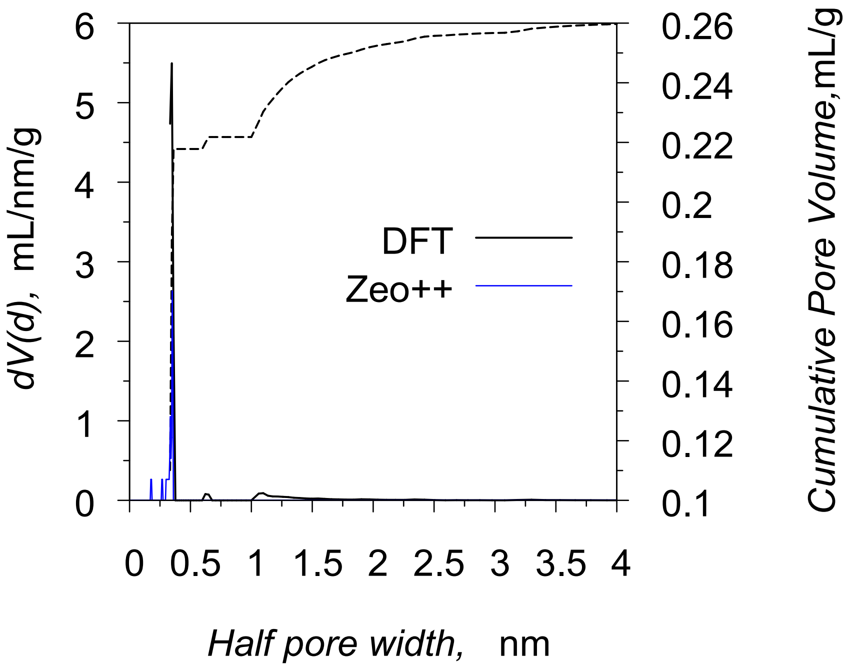

Figure S8. Pore size distributions for **1** calculated by DFT (N_2 adsorption at 77 K) and Zeo++ [1, 2].

Gas adsorption isotherms at 273 K and 298 K were fitted by virial equation (S1) in order to calculate Henry constants and isosteric heats of adsorption.

$$\ln p = \ln n + \frac{1}{T}\sum_i A_i \cdot n^i + \sum_j B_j \cdot n^j \quad \text{(S1)}$$

Virial coefficients are summarized in Table S2, whereas fit plots are shown in Figure S6.

Table S2. Virial coefficients A_i and B_i for gas adsorption isotherms at 273 K and 298 K on **1.**

Gas	Coefficients
CO_2	A0 = -3134.2<br>A1 = 114.639<br>B0 = 9.42998
CH_4	A0 = -2375.36<br>A1 = 124.085<br>B0 = 8.22832
N_2	A0 = -1061.3<br>B0 = 5.41696
O_2	A0 = -1236.34<br>B0 = 6.29766<br>B1 = 0.622471
CO	A0 = -2079.3<br>A1 = 189.31

	B0	= 8.60369

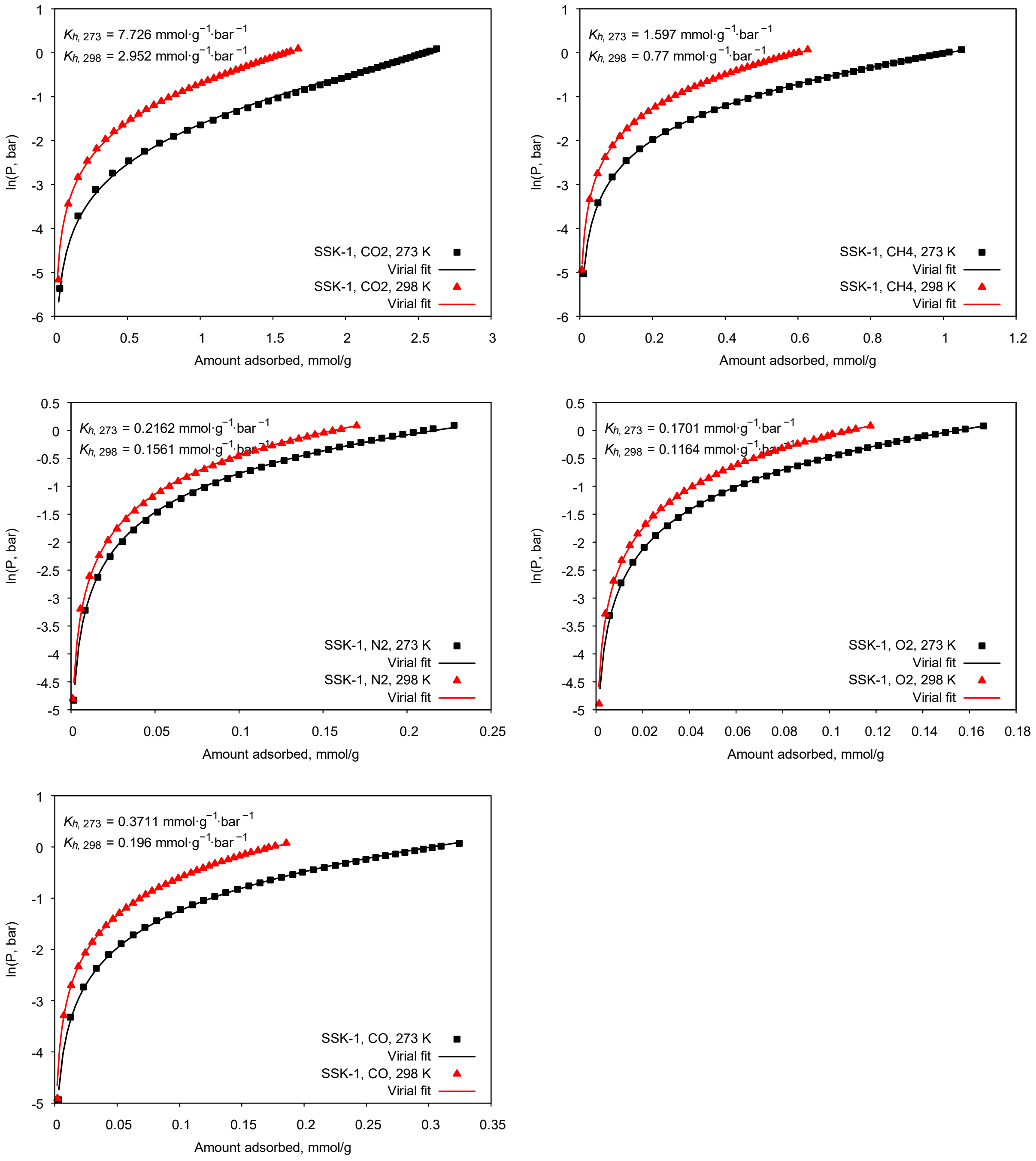

Figure S9. Fits of isotherms by virial equations.

Heats of adsorption

Isosteric heats of adsorption were calculated using virial coefficients by equation (S2):

$$\Delta H^{\circ} = R \cdot \sum_{i} A_i \cdot n^i \tag{S2}$$

The corresponding graphs are shown in Figure S7.

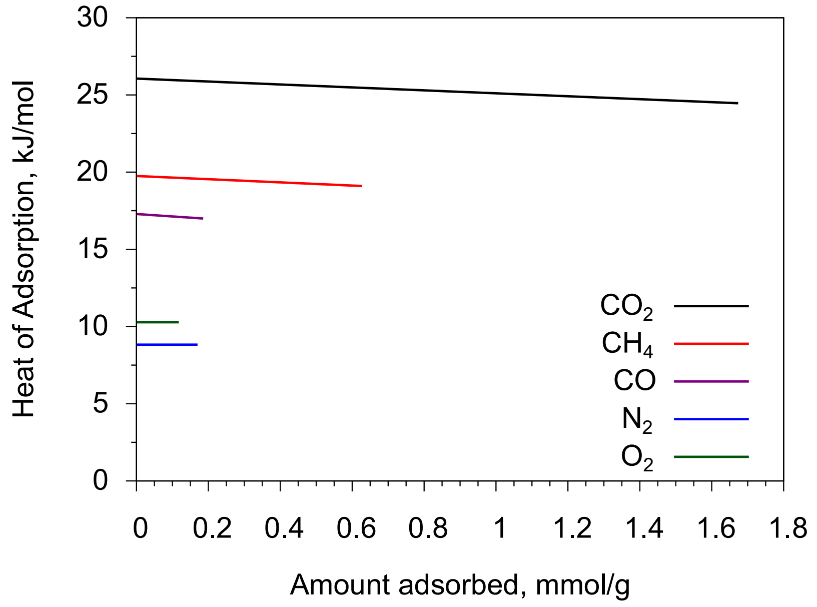

Figure S10. Isosteric heats of gas adsorption on **1**.

Henry constants

Henry constants were calculated using virial coefficients by equation (S3):

$$K_h = exp\left[\frac{-A_0}{T} - B_0\right] \tag{S3}$$

Table S3. Henry constants for gas adsorption on **1** in mmol·g^{-1}·bar^{-1} at 273 K and 298 K.

**Gas\Temperature**	**273 К**	**298 К**
CO_2	7.726	2.952
CH_4	1.597	0.770
N_2	0.2162	0.1561
O_2	0.1701	0.1164
CO	0.3711	0.1960

Fit of adsorption isotherms

Adsorption isotherms were fitted by the most appropriate model for IAST calculations. Fittings were performed for isotherms in mL/g–torr units, so parameters are in the corresponding units.

CO_2: Langmuir−Freundlich model

CH_4: Langmuir model

N_2: Langmuir model

O_2: Langmuir model

CO: Langmuir model

Fitted parameters are summarized in Table S4. Fitted isotherms are shown in Figure S8.

Table S4. Fitted parameters for adsorption isotherms on **1** at 273 K and 298 K

**Gas**	**273 K**	**298 K**
CO_2	w = 88.4914<br>b = 0.00217916<br>t = 0.985349	w = 86.0091<br>b = 0.000980014<br>t = 1.00567
CH_4	w = 63.1814<br>b = 0.000741005	w = 57.9179<br>b = 0.000399736
N_2	w = 60.0168<br>b = 0.000113724	w = 165.73<br>b = 2.89476e-05
O_2	w = 33.4228<br>b = 0.000154405	w = 45.9629<br>b = 7.47483e-05
CO	w = 46.2654<br>b = 0.000232935	w = 35.8648<br>b = 0.000162124

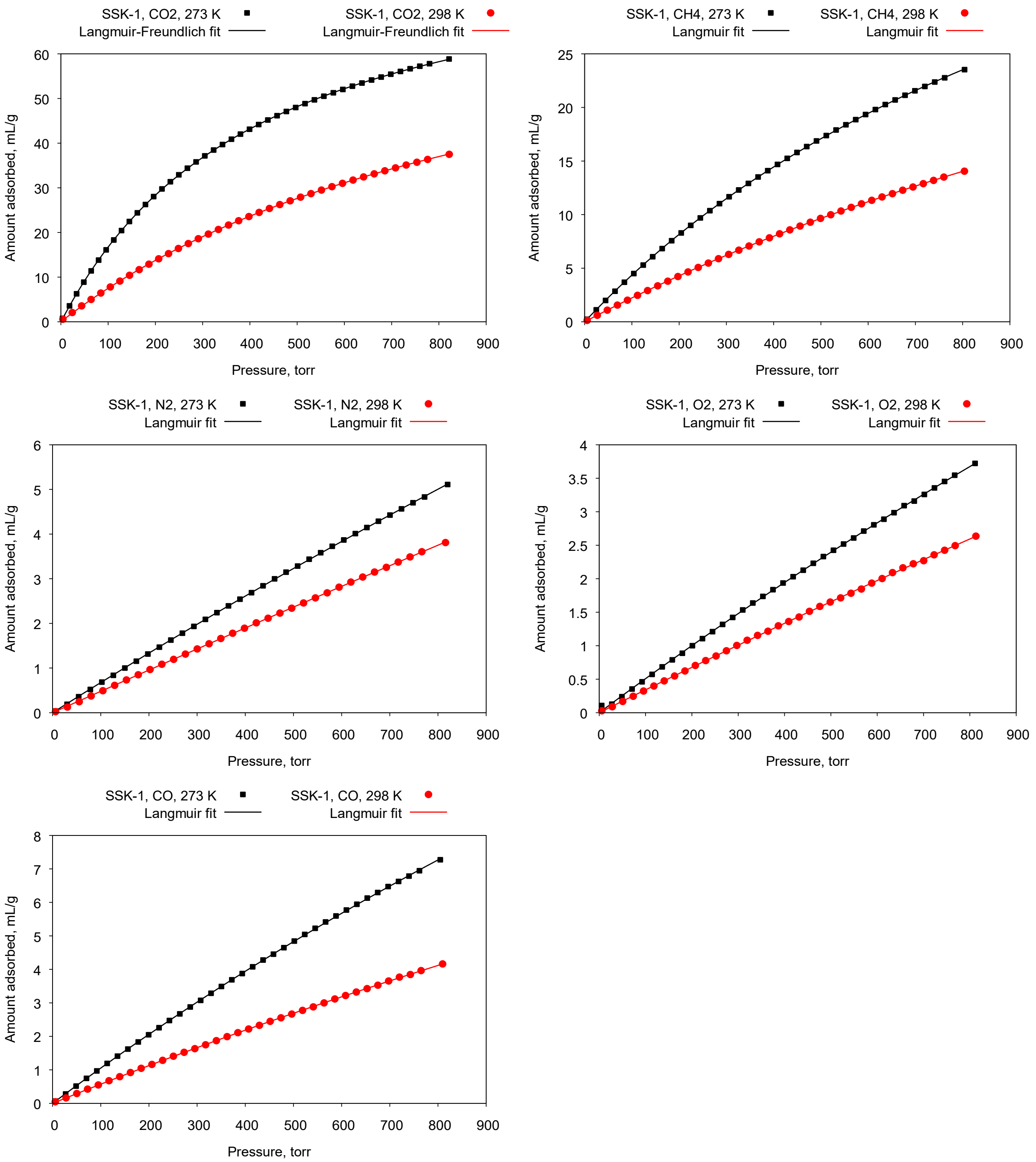

Figure S11. Fits of the gas adsorption isotherms by appropriate models.

Selectivity calculations

Selectivity factors were evaluated by three commonly used methods:

(i) As the molar ratio of the adsorption quantities at the relevant partial pressures of the gases:

$$S = \frac{n_1/n_2}{p_1/p_2}, \tag{S2}$$

where S is the selectivity factor, n_i represents the adsorbed amount of component i, and p_i represents the partial pressure of component i.

(ii) As a ratio of Henry constants which corresponds to the slope of the adsorption isotherm at very low partial pressures:

$$S = \frac{K_{H1}}{K_{H2}} \tag{S3}$$

(iii) By ideal adsorbed solution theory (IAST). The relationship between P, y_i and x_i (P — the total pressure of the gas phase, y_i — mole fraction of the i-component in gas phase, x_i — mole fraction of the i-component in absorbed state) is defined according to the IAST theory [3]:

$$\int_{p=0}^{p=\frac{Py_1}{x_1}} n_1(p)dlnp = \int_{p=0}^{p=\frac{Py_2}{x_2}} n_2(p)dlnp \tag{S4}$$

In this case the selectivity factors were determined as:

$$S = \frac{y_2/x_2}{y_1/x_1} = \frac{x_1(1-y_1)}{y_1(1-x_1)} \tag{S5}$$

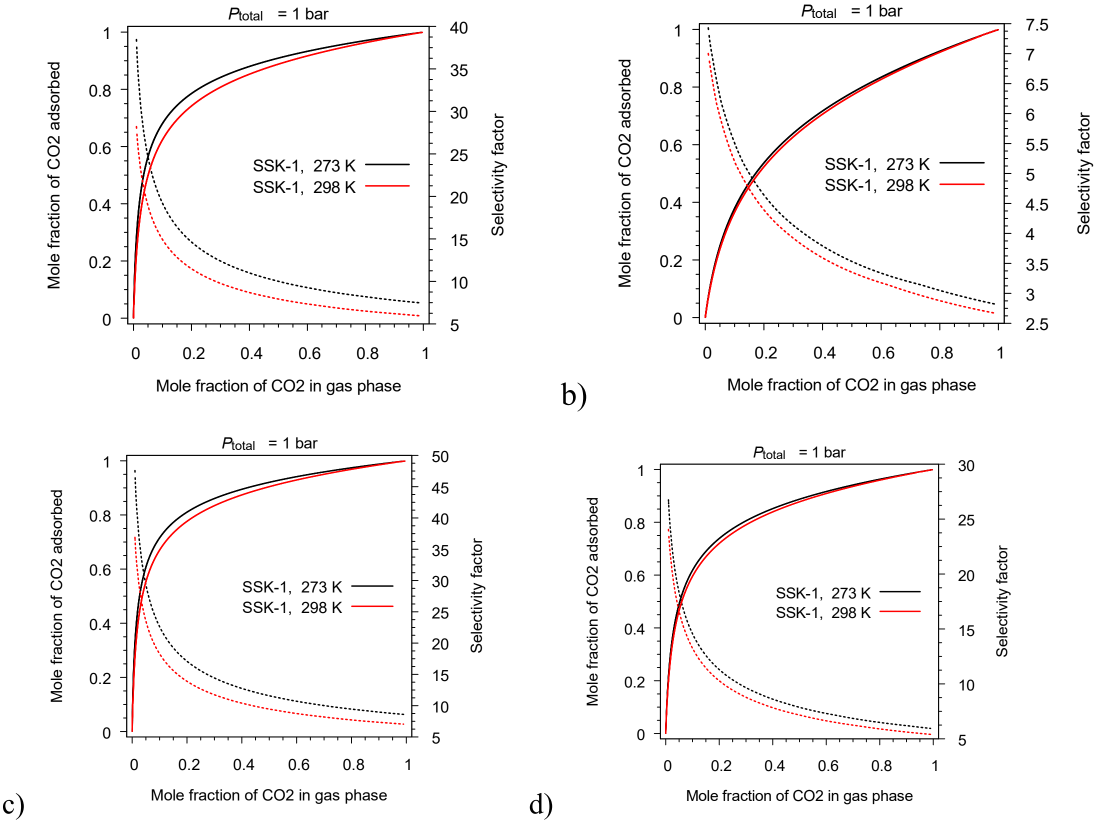

Fig. S12. Prediction of adsorption equilibrium by IAST (solid lines) and dependence of selectivity factors on gas phase composition (dashed lines) for binary gas mixtures: a) CO_2/N_2; b) CO_2/CH_4; c) CO_2/O_2; d) CO_2/CO.

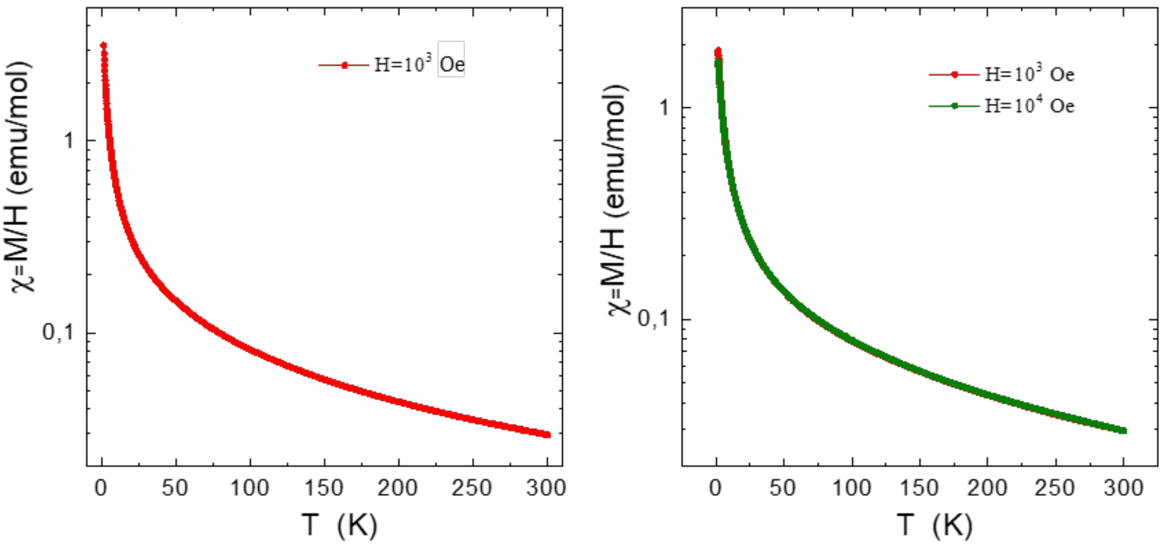

Fig. S13. Temperature dependences of the magnetic susceptibility for compounds **1** (left) and **2** (right). The magnetic susceptibility of both compounds exhibits dependence neither on magneto-thermal history, nor on the magnetic field value, except for the lowest temperatures where the curves measured at 1 and 10 kOe diverge slightly due to nonlinear $M(H)$ dependence.

References:

1. Willems, T.F.; Rycroft, C.H.; Kazi, M. ; Meza, J.C.; Haranczyk, M. Algorithms and Tools for High-Throughput Geometry-Based Analysis of Crystalline Porous Materials. *Microporous Mesoporous Mater.* **2012**, *149*, 134-141.
2. Pinheiro, M.; Martin, R.L.; Rycroft, C.H.; Jones, A.; Iglesia, E.; Haranczyk, M. Characterization and Comparison of Pore Landscapes in Crystalline Porous Materials. *J. Mol. Graph. Model.* **2013**, *44* (2013) 208-219.
3. Myers, A.L.; Prausnitz, J.M.; Thermodynamics of Mixed-Gas Adsorption. *AIChE J.*, **1965**, 11, 121–127.
