# Supplementary material for: 3D Metal–Organic Frameworks Based on Co(II) and Bithiophendicarboxylate: Synthesis, Crystal Structures, Gas Adsorption, and Magnetic Properties
Source: Molecules. 2021 Feb 26;26(5):1269. doi: 10.3390/molecules26051269 (PMC7956319; doi:10.3390/molecules26051269)
Supplement: Supplementary file 1 [file molecules-26-01269-s001.zip › SI.doc]

**3D metal-organic frameworks based on Co(II) and bithiophendicarboxylate: synthesis, crystal structures, gas adsorption, and magnetic properties**

Vadim A. Dubskikh, Anna A. Lysova, Denis G. Samsonenko, Alexander N. Lavrov, Konstantin A. Kovalenko, Danil N. Dybtsev,* Vladimir P. Fedin

Nikolaev Institute of Inorganic Chemistry, Siberian Branch of the Russian Academy of Sciences, 3 Acad. Lavrentiev Ave., 630090, Novosibirsk, Russia

*Corresponding author, dan@niic.nsc.ru

Table S1. Crystal data and structure refinement for **1–3**.

| Identification code | **1** | **2** | **3** |  |
| --- | --- | --- | --- | --- |
| Empirical formula | C_62_H_56_N_8_O_16_S_6_Co_3_ | C_52_H_61_N_8_O_19.5_S_6_Co_3_ | C_48_H_58_N_6_O_20_S_6_Co_3_ |  |
| *M*, g/mol | 1528.29 | 1479.23 | 1408.15 |  |
| *T, K* | 100 | 100 | 100 |  |
| Crystal system | *Monoclinic* | *Orthorhombic* | *Monoclinic* |  |
| Space group | *P*2_1_/*c* | *Fddd* | *P*2_1_/*c* |  |
| *a*, Å | 10.852(2) | 11.746(2) | 11.96(2) |  |
| *b*, Å | 25.406(5) | 42.150(4) | 25.867(8) |  |
| *c*, Å | 12.801(2) | 51.849(2) | 11.461(16) |  |
| α, град. | 90 | 90 | 90 |  |
| β, град. | 109.74(3) | 90 | 106.49(3) |  |
| γ, degree | 90 | 90 | 90 |  |
| *V*, Å^3^ | 3322.0(12) | 25670(5) | 3400(8) |  |
| *Z* | 2 | 16 | 2 |  |
| *D*(calc.), g/cm^3^ | 1.538 | 1.531 | 1.376 |  |
| μ, mm^−1^ | 1.345 | 1.392 | 1.309 |  |
| *F*(000) | 1578 | 12192 | 1450 |  |
| Crystal size, mm | 0.15 × 0.15 × 0.05 | 0.10 × 0.05 × 0.05 | 0.05 × 0.05 × 0.02 |  |
| θ range for data collection, deg. | 2.40–30.98 | 1.39–31.03 | 2.58–32.41 |  |
| Index ranges | −13 < *h* < 13,  −28 < *k* < 32,  −16 < *l* < 16 | −15 < *h* < 15,  −54 < *k* < 54,  −59 < *l* < 64 | −14 < *h* < 14,  −33 < *k* < 33,  −15 < *l* < 15 |  |
| Reflections collected / independent | 19683 / 7317 | 50295 / 7189 | 28176 / 7481 |  |
| *R*_int_ | 0.0514 | 0.0585 | 0.0782 |  |
| Reflections with *I* > 2σ(*I*) | 6412 | 6073 | 4702 |  |
| Goodness-of-fit on *F*^2^ | 1.052 | 1.073 | 1.044 |  |
| Final *R* indices [*I* > 2σ(*I*)] | *R*_1_ = 0.0506,  *wR*_2_ = 0.1375 | *R*_1_ = 0.0361, *wR*_2_ = 0.0961 | *R*_1_ = 0.0764,  *wR*_2_ = 0.2142 |  |
| *R* indices (all data) | *R*_1_ = 0.0575,  *wR*_2_ = 0.1429 | *R*_1_ = 0.0442 *wR*_2_ = 0.0997 | *R*_1_ = 0.1170,  *wR*_2_ = 0.2365 |  |
| Largest diff. peak / hole, *e*/Å^3^ | 0.723 / −0.738 | 0.390 / –0.517 | 0.863 / −0.875 |  |

**Figure S1.** Coordination environment of Co(II) cations in **1** (а) and **3** (b). a) Ellipsoids of 50% probability. Symmetry code: i): 1 - x, 1 - y, 1 – z; ii) x, ½ - y, ½ + z; iii) 1 – x, ½ + y, ½ - z. b) Ellipsoids of 30% probability. Symmetry code: i): 1 - x, 1 - y, 1 – z; ii) 1 - x, ½ + y, 3/2 - z; iii) x, ½ - y, z - ½. Only one of the possible orientations of coordinated DMF molecules and btdc^2-^ ligands is shown. Hydrogen atoms are not shown.

**Figure S2.** Coordination environment of Co(II) cations in **2**. Ellipsoids of 50% probability. Hydrogen atoms are not shown. Symmetry code (i): 1/2 - x, 1 - y, 3/2 - z.

**Figure S3**. Powder X-ray diffraction patterns for compounds **1** and **2** (experimentally observed – in blue and red, respectively, theoretical – in light-blue and pink, respectively).

**Figure S4**. The IR spectrum of the compound **1**.

**Figure S5**. The IR spectrum of the compound **2**.

### Isotherms

**Figure S6.** Semilogarithmic representation of nitrogen adsorption (filled squares) and desorption (open squares) isotherms at 77 K for the compound **1**.

(a) (b)

**Figure S7.** Nitrogen adsorption isotherm fit (from pore size distribution calculation): a) normal scale; b) semilogarithmic scale.

**Figure S8.** Pore size distributions for **1** calculated by DFT (N_2_ adsorption at 77 K) and Zeo++ [1, 2].

Gas adsorption isotherms at 273 K and 298 K were fitted by virial equation (S1) in order to calculate Henry constants and isosteric heats of adsorption.

|  | (S1) |
| --- | --- |

Virial coefficients are summarized in Table S2, whereas fit plots are shown in Figure S6.

**Table** **S2**. Virial coefficients *A_i_* and *B_i_* for gas adsorption isotherms at 273 K and 298 K on 1***.***

| Gas | Coefficients |
| --- | --- |
| CO_2_ | A0 = -3134.2  A1 = 114.639  B0 = 9.42998 |
| CH_4_ | A0 = -2375.36  A1 = 124.085  B0 = 8.22832 |
| N_2_ | A0 = -1061.3  B0 = 5.41696 |
| O_2_ | A0 = -1236.34  B0 = 6.29766  B1 = 0.622471 |
| CO | A0 = -2079.3  A1 = 189.31  B0 = 8.60369 |

**Figure S9.** Fits of isotherms by virial equations.

### Heats of adsorption

Isosteric heats of adsorption were calculated using virial coefficients by equation (S2):

|  | (S2) |
| --- | --- |

The corresponding graphs are shown in Figure S7.

**Figure S10**. Isosteric heats of gas adsorption on **1**.

### Henry constants

Henry constants were calculated using virial coefficients by equation (S3):

|  | (S3) |
| --- | --- |

**Table S3**. Henry constants for gas adsorption on 1 in mmol·g^−1^·bar^−1^ at 273 K and 298 K.

| Gas\Temperature | 273 К | 298 К |
| --- | --- | --- |
| CO_2_ | 7.726 | 2.952 |
| CH_4_ | 1.597 | 0.770 |
| N_2_ | 0.2162 | 0.1561 |
| O_2_ | 0.1701 | 0.1164 |
| CO | 0.3711 | 0.1960 |

### Fit of adsorption isotherms

Adsorption isotherms were fitted by the most appropriate model for IAST calculations. Fittings were performed for isotherms in mL/g–torr units, so parameters are in the corresponding units.

CO_2_: Langmuir−Freundlich model

CH_4_: Langmuir model

N_2_: Langmuir model

O_2_: Langmuir model

CO: Langmuir model

Fitted parameters are summarized in Table S4. Fitted isotherms are shown in Figure S8.

**Table** **S4**. Fitted parameters for adsorption isotherms on 1 at 273 K and 298 K

| Gas | 273 K | 298 K |
| --- | --- | --- |
| CO_2_ | w = 88.4914  b = 0.00217916  t = 0.985349 | w = 86.0091  b = 0.000980014  t = 1.00567 |
| CH_4_ | w = 63.1814  b = 0.000741005 | w = 57.9179  b = 0.000399736 |
| N_2_ | w = 60.0168  b = 0.000113724 | w = 165.73  b = 2.89476e-05 |
| O_2_ | w = 33.4228  b = 0.000154405 | w = 45.9629  b = 7.47483e-05 |
| CO | w = 46.2654  b = 0.000232935 | w = 35.8648  b = 0.000162124 |

**Figure S11.** Fits of the gas adsorption isotherms by appropriate models.

### Selectivity calculations

Selectivity factors were evaluated by three commonly used methods:

(i) As the molar ratio of the adsorption quantities at the relevant partial pressures of the gases:

| , | (S2) |
| --- | --- |

where *S* is the selectivity factor, *n_i_* represents the adsorbed amount of component *i*, and *p_i_* represents the partial pressure of component *i*.

(ii) As a ratio of Henry constants which corresponds to the slope of the adsorption isotherm at very low partial pressures:

|  | (S3) |
| --- | --- |

(iii) By ideal adsorbed solution theory (IAST). The relationship between *P*, *y_i_* and *x_i_* (*P* — the total pressure of the gas phase, *y_i_* — mole fraction of the *i‑*component in gas phase, *x_i_* — mole fraction of the *i*-component in absorbed state) is defined according to the IAST theory [3]:

|  | (S4) |
| --- | --- |

In this case the selectivity factors were determined as:

|  | (S5) |
| --- | --- |

 b) 

c)d)

**Fig. S12.** Prediction of adsorption equilibrium by IAST (solid lines) and dependence of selectivity factors on gas phase composition (dashed lines) for binary gas mixtures: a) CO_2_/N_2_; b) CO_2_/CH_4_; c) CO_2_/O_2_; d) CO_2_/CO.

**Fig. S13**. Temperature dependences of the magnetic susceptibility for compounds **1** (left) and **2** (right). The magnetic susceptibility of both compounds exhibits dependence neither on magneto-thermal history, nor on the magnetic field value, except for the lowest temperatures where the curves measured at 1 and 10 kOe diverge slightly due to nonlinear *M*(*H*) dependence.

**References:**

1. Willems, T.F.; Rycroft, C.H.; Kazi, M. ; Meza, J.C.; Haranczyk, M. Algorithms and Tools for High-Throughput Geometry-Based Analysis of Crystalline Porous Materials. *Microporous Mesoporous Mater.* **2012**, *149*, 134-141.
2. Pinheiro, M.; Martin, R.L.; Rycroft, C.H.; Jones, A.; Iglesia, E.; Haranczyk, M. Characterization and Comparison of Pore Landscapes in Crystalline Porous Materials. *J. Mol. Graph. Model.* **2013**, *44* (2013) 208-219.
3. Myers, A.L.; Prausnitz, J.M.; Thermodynamics of Mixed-Gas Adsorption. *AIChE J.*, **1965**, 11, 121–127.
